# Treatment of post-vaccination optic neuritis: implications from the global SARS-CoV-2 vaccination effort

**DOI:** 10.1007/s00417-025-06805-w

**Published:** 2025-11-29

**Authors:** Yan Ning Neo, Lidia Martinez-Alvarez, Indran Davagnanam, Gabriela Girafa, Fion Bremner, Tasanee Braithwaite, Zhaleh Khaleeli, Victoria Nowak, Ahmed T. Toosy, Axel Petzold

**Affiliations:** 1 Moorfields Eye Hospital NHS Foundation Trust, 162 City Road, London, EC1V 2PD UK; 2 Birmingham and Midland Eye Centre, Sandwell and West, Birmingham Hospitals NHS Trust, Birmingham, UK; 3 Queen Elizabeth Hospital Birmingham, UniversityHospitalsBirmingham, Birmingham, UK; 4 National Hospital for Neurology and Neurosurgery, University College London Hospitals NHS Foundation Trust, London, UK Queen Square,; 5 King’s Health Partners Centre for Translational Medicine, London, UK; 6 Guy’s and St Thomas’ NHS Foundation Trust, London, UK; 7 Dept. Of Neuroinflammation, Queen Square MS CentreUCLSquare Institute of NeurologyUniversity College London, London, UK

**Keywords:** Optic neuritis, Vaccination, Treatment, Outcome

## Abstract

**Purpose:**

Optic neuritis (ON) is a rare but treatable side effect of vaccination. The global vaccination effort to SARS-CoV-2 offered a unique chance to study post-vaccination ON.

**Methods:**

A one-year, prospective, multi-center follow-up study by the International Consortium for ON (ICON) in specialized neuro-ophthalmological clinical centers. The pre-specified protocol was confirm with the international consensus diagnostic criteria for making a diagnosis of definite ON. The main outcome measures were speed of treatment initiation, best corrected high contrast visual acuity and retinal asymmetry metrics on optical coherence tomography indicating atrophy.

**Results:**

Inclusion of 73 individuals from 15 countries with ON following SARS-CoV-2 vaccination. There were notable differences in characteristics of post-vaccination ON meeting diagnostic criteria compared to pre-COVID19 pandemic incidence studies. These included more frequent bilateral presentation 17/69 (25%) and older age at onset. Post-vaccination ON mostly manifested after the first vaccine dose in 58 individuals (84%). The most frequent (78%) presenting symptom was pain which worsened on eye movements. Twenty-two percent had autoantibodies to MOG and none to AQP4. Initial median visual acuity was logMAR 1.0, improving to logMAR 0.0 at one-year follow-up. Early corticosteroid treatment significantly preserved retinal nerve fiber, macular ganglion cell, and inner nuclear layers compared to later or no treatment.

**Conclusion:**

Functional visual recovery in post-vaccination ON cases is good. Rapid treatment with corticosteroids provides neuroprotection, underscoring the need for timely intervention. Long-term management depends on presence of autoantibodies.

**Key messages:**

***What is known***

- Post-vaccination optic neuritis (ON) was previously considered a rare, anecdotal occurrence before the COVID-19 pandemic.
- Retrospective database studies conducted after the pandemic identified ON as the second most common vaccine-related complication affecting vision.

***What is new***

- A prospective, deeply phenotyped global cohort of post-vaccination ON was studied using a pre-specified protocol and novel consensus diagnostic criteria.
- Longitudinal data demonstrated excellent recovery of visual function, despite measurable atrophy in the retinal nerve fibre layer (RNFL) and ganglion cell layer (GCL) on optical coherence tomography (OCT).
- Evidence supports that rapid initiation of corticosteroid treatment helps preserve the RNFL and GCL, highlighting this as a treatable condition with favorable outcomes when managed promptly.

**Supplementary Information:**

The online version contains supplementary material available at 10.1007/s00417-025-06805-w.

## Introduction

The global vaccination campaign against SARS-CoV-2 has provided a unique opportunity to systematically investigate rare clinical events that would otherwise be extremely difficult to study. The well-documented nature of these vaccinations has facilitated prospective analyses of potential side effects, which is crucial not only for understanding the implications of the SARS-CoV-2 vaccine but also for enhancing our overall approach to vaccine side effects.

This paper discusses the evolution of clinical observations of a significant cluster of severe bilateral optic neuritis (ON) in elderly patients coinciding with the commencement of SARS-CoV-2 vaccinations into a global, prospective study by the International Consortium for ON (ICON). Expanding on our initial baseline findings (only published as pre-print) [1] subsequent retrospective database studies have identified ON as the second most frequent visual complication post-vaccination [2, 3] For 1,000,000 vaccinated individuals, 6.6 post vaccination events were recorded, of which anterior uveitis was most frequent (58.3%), followed by optic neuritis (39.3%) [2]. However, a notable limitation of these retrospective studies is their lack of detailed, case-specific information. In clinical practice, accurate recognition of clinical phenotypes is essential for guiding appropriate investigations and ensuring safe patient management. Our study seeks to bridge this gap in current literature.

## Methods

This study, approved by the Institutional Research Board at Moorfields Eye Hospital (study number CaRS_24), involved a collaborative effort with the ICON study group, consisting of experts from 55 countries (contributors marked with an asterisk in the Acknowledgment). The protocol was pre-specified, mandating all clinical and para-clinical tests for making a diagnosis of definite ON based on a manuscript which was *in print* at the time [4], but known to the group. We analyzed data on optic neuritis post SARS-CoV-2 vaccination in adults, from 15 countries, between February 2021 and December 2021. We also collected post-infectious ON following SARS-CoV-2 between March 2020 and December 2021. A positive COVID-19 test at presentation was mandatory to separate post-infectious (COVID-19 test result positive) from post-vaccination (COVID-19 test result negative) ON.

### Inclusion criteria

Two ophthalmologists (LMA, FB) and three neurologists (ZK, AP, ATT) reviewed all 73 patients submitted by ICON members, applying the 2022 ICON consensus diagnostic criteria for definite ON and possible ON [4]. All COVID-19 vaccines available at the time qualified for inclusion.

### Exclusion criteria

Cases not meeting the ICON 2022 diagnostic criteria were excluded. Likewise patients meeting the consensus criteria for post-infectious ON [4] were excluded. The ICON group only identified 4 cases of post-infectious (COVID-19) ON during the observation period.

### Patient assessment

For post-vaccination ON, a time delay of less than 28 days indicated a strong association, while 28 days to 3 months suggested a weak association. The ON phenotype were further classified into MOG-associated, MS-associated, and AQP4-associated ON based on MRI and biomarker data [4]. The timing of ON was categorized as acute (0–7 days) or subacute (7 days to 3 months) from onset of symptoms. Ethnicity reported included Asian (including Indian and Middle Eastern), African (including Afrocaribbean), Caucasian, and Hispanic.

## Paraclinical tests

### Optical Coherence Tomography (OCT)

Retinal images were obtained using state of the art spectral domain OCT (Heidelberg Spectralis, Zeiss Cirrus, Topcon 1000/Triton) and fundus photography (Topcon TRC-50DX). For OCT, a 3.4 mm diameter ring scan around the optic disc was taken to report the peripapillary retinal nerve fibre (pRNFL) thickness (Spectralis OCT) and for Cirrus and Topcon devices these data were extracted from optic disc volume scans. A macular volume scan was taken to calculate the thickness of the combined ganglion and inner plexiform layers (mGCIPL). Metrics of retinal asymmetry were calculated over time as described [6]. An inter-eye difference of > 4% for the mGCIPL or > 5% for the pRNFL at least 3 months after onset of ON was required to meet the diagnostic criteria [4]. Neurodegeneration was defined as atrophy at follow-up compared to baseline. Neuroprotection was defined based on this metric as reduced atrophy in between groups. All OCT scans were reviewed by one neuro-ophthalmologist (YNN) and one neurologist (AP). The OSCAR-IB criteria were used for OCT quality control [7] and data reporting followed the APOSTEL 2.0 guidelines (see also supplementary Table 1) [8].

### Magnetic Resonance Imaging (MRI)

All MRI scans were assessed in each expert center and the report included to Table 1. For the UK patients only, MRI scans were reviewed and scored by two experienced neuro-radiologists (KM, ID) and one neurologist (ATT) for diagnostic confirmation of ON. The raters were blinded to all other data. Optic nerve lesion lengths on STIR (short tau inversion recovery) and post-gadolinium sequences were calculated, and enhancement, swelling, adnexal involvement and intracranial pathology, especially of inflammatory/demyelinating lesions were determined.5 Discrepancies were resolved by consensus opinion. For non-UK patients, MRI reports were used to confirm radiological ON.

**Table 1:** Baseline description of the cohort and summary of the diagnostic work-up, diagnostic categories, treatment and outcomes. (AZ = AstraZeneca; JA = Janssen; PZ = Pfizer-BioNTech; SN = Sinovac). OCT Inter-eye difference = IED. Data are presented as median (range) and numbers (percentages within column)

		AZ	JA	PZ	SN	Total
Patients N (%)		43 (62%)	1 (1%)	20 (29%)	5 (7%)	69 (100%)
Vaccination history	First vaccine	38 (88%)	1 (100%)	16 (80%)	3 (60%)	58 (84%)
	Second vaccine	5 (12%)	0 (0%)	4 (20%)	2 (40%)	11 (16%)
‍COVID-19 test	positive	0 (0%)	0 (0%)	0 (0%)	0 (0%)	0 (0%)
Gender	Female	28 (65%)	1 (100%)	13 (65%)	5 (100%)	47 (68%)
	Male	15 (35%)	0 (0%)	7 (35%)	0 (0%)	22 (32%)
Age	(years)	48 (18–75)	37 (37)	42 (22–79)	42 (21–50)	45 (18–79)
Ethnicity	Caucasian	34 (79%)	1 (100%)	19 (95%)	4 (80%)	58 (84%)
	Asian	3 (7%)	0 (0%)	1 (5%)	1 (20%)	5 (7%)
	Afro-carribean	3 (7%)	0 (0%)	0 (0%)	0 (0%)	3 (4%)
	Hispanic	3 (7%)	0 (0%)	0 (0%)	0 (0%)	3 (4%)
Time to onset	(days)	20 (1–69)	10 (10)	14 (1–45)	10 (7–14)	16 (1–69)
Laterality	Unilateral	30 (70%)	1 (100%)	16 (80%)	5 (100%)	52 (75%)
	Bilateral	13 (30%)	0 (0%)	4 (20%)	0 (0%)	17 (25%)
RAPD	Present	33 (77%)	0 (0%)	16 (80%)	5 (100%)	54 (78%)
	Absent	8 (19%)	1 (100%)	3 (15%)	0 (0%)	12 (17%)
Pain at onset	yes	35 (81%)	1 (100%)	14 (70%)	4 (80%)	54 (78%)
	no	7 (16%)	0 (0%)	4 (20%)	1 (20%)	12 (17%)
Disc swelling	yes	27 (63%)	1 (100%)	10 (50%)	2 (40%)	40 (58%)
	no	16 (37%)	0 (0%)	10 (50%)	3 (60%)	29 (42%)
OCT IED	positive	25 (58%)	1 (100%)	11 (55%)	2 (40%)	39 (57%)
	negative	18 (42%)	0 (0%)	9 (45%)	3 (60%)	30 (43%)
MRI	positive	37 (86%)	1 (100%)	15 (75%)	4 (80%)	57 (83%)
	normal	6 (14%)	0 (0%)	5 (25%)	1 (20%)	12 (17%)
Anti MOG antibody	positive	13 (30%)	0 (0%)	2 (10%)	1 (20%)	16 (23%)
	negative	27 (63%)	1 (100%)	16 (80%)	3 960%)	47 (68%)
	not available	3 (7%)	0 (0%)	2 (10%)	1 (20%)	6 (9%)
Aquaporin 4 (AQP4) antibody	positive	0 (0%)	0 (0%)	0 (0%)	0 (0%)	0 (0%)
	negative	40 (93%)	1 (100%)	18 (90%)	4 (80%)	63 (91%)
	not available	3 (7%)	0 (0%)	2 (10%)	1 (20%)	6 (9%)
Subtype of optic neuritis	SION	26 (60%)	0 (0%)	12 (60%)	4 (80%)	42 (61%)
	MOG-ON	12 (28%)	0 (0%)	2 (10%)	1 (20%)	15 (22%)
	MS-ON	5 (12%)	1 (100%)	6 (30%)	0 (0%)	12 (17%)
Methylprednisolone	intravenous	27 (63%)	1 (100%)	14 (70%)	5 (100%)	47 (68%)
	oral	13 (30%)	0 (0%)	2 (10%)	0 (0%)	15 (22%)
PLEX		3 (7%)	0 (0%)	4 (20%)	0 (0%)	7 (10%)
Time from onset to treatment	(days)	9 |(2–120)	6 (6)	8 (2–30)	8 (3–37)	8 (2–120)
Time from treatment to analgesia	(hours)	36 (6–240)	0 (0)	48 (9–168)	24 (24)	36 (6–240)
BCVA at onset (LogMAR)		0.8 (−0.1–3)	0 (0)	0.5 (0–3)	1.9 (0–1.9)	0.7 (−0.1–3.0)
BCVA after treatment (LogMAR)		0 (−0.2–3)	0 (0)	0 (0–1.9)	0 (0–3)	0–7 (−0.1–3.0)

### Biomarker

Autoantibodies for Aquaporin 4 (AQP4) and Myelin oligodendrocyte glycoprotein (MOG) were advised to be determined by state-of-the-art cell based assays [4, 9–11] Isoelectric Focusing was used to provide evidence for intrathecal IgG synthesis, also referred to as oligoclonal bands. [12]

### Outcome measures

The primary outcome measure was high contrast visual acuity. The secondary outcome measure was a metric of retinal asymmetry, the inter-eye percentage difference (IEPD) of the thickness of the mGCIPL [6, 13, 14]. The mGCIPL was reported to be superior to the pRNFL regarding diagnostic sensitivity and specificity, and is less prone to swelling of axons [4]. The use of the IEPD also overcomes the limitation of absolute pRNFL and mGCIPL thickness values not being directly comparable between the three different OCT devices used in this study.

### Statistical Analysis

The data distribution was analysed visually and using the Shapiro–Wilk test using SAS (v9.4m7, 100 SAS Campus DriveCary, NC 27513–2414, US). The median and range were shown for non-Gaussian data. Categorical data were presented as numbers and percentages (decimals rounded to natural numbers). Parametric tests were used for Gaussian data and non-parametric tests for non-Gaussian and unbalanced data. General linear models were used for comparison of more than two groups. Spearman’s correlation coefficient was used for non-Gaussian data. For individuals with bilateral involvement the VA of the worst affected eye was used. For unilateral cases the VA of the affected eye was used. We provided the exact number of data points for each analysis and variable to account for incomplete observations. The null hypothesis was rejected if p < 0.05. All analyses were performed for patients meeting the ICON 2022 diagnostic criteria for definite ON [4]. This was followed by a sensitivity analysis including the patients with a diagnosis of possible ON [4].The effect of treatment was assessed by treatment modality and speed of treatment initiation in days.

### Data sharing statement

The annonymized data collected for the study, including individual participant data and a data dictionary defining each field in the set, will be made available to others upon reasonable request. The data can be requested by email from the corresponding author.

## Results

### Patient characteristics

**Fig. 1:**
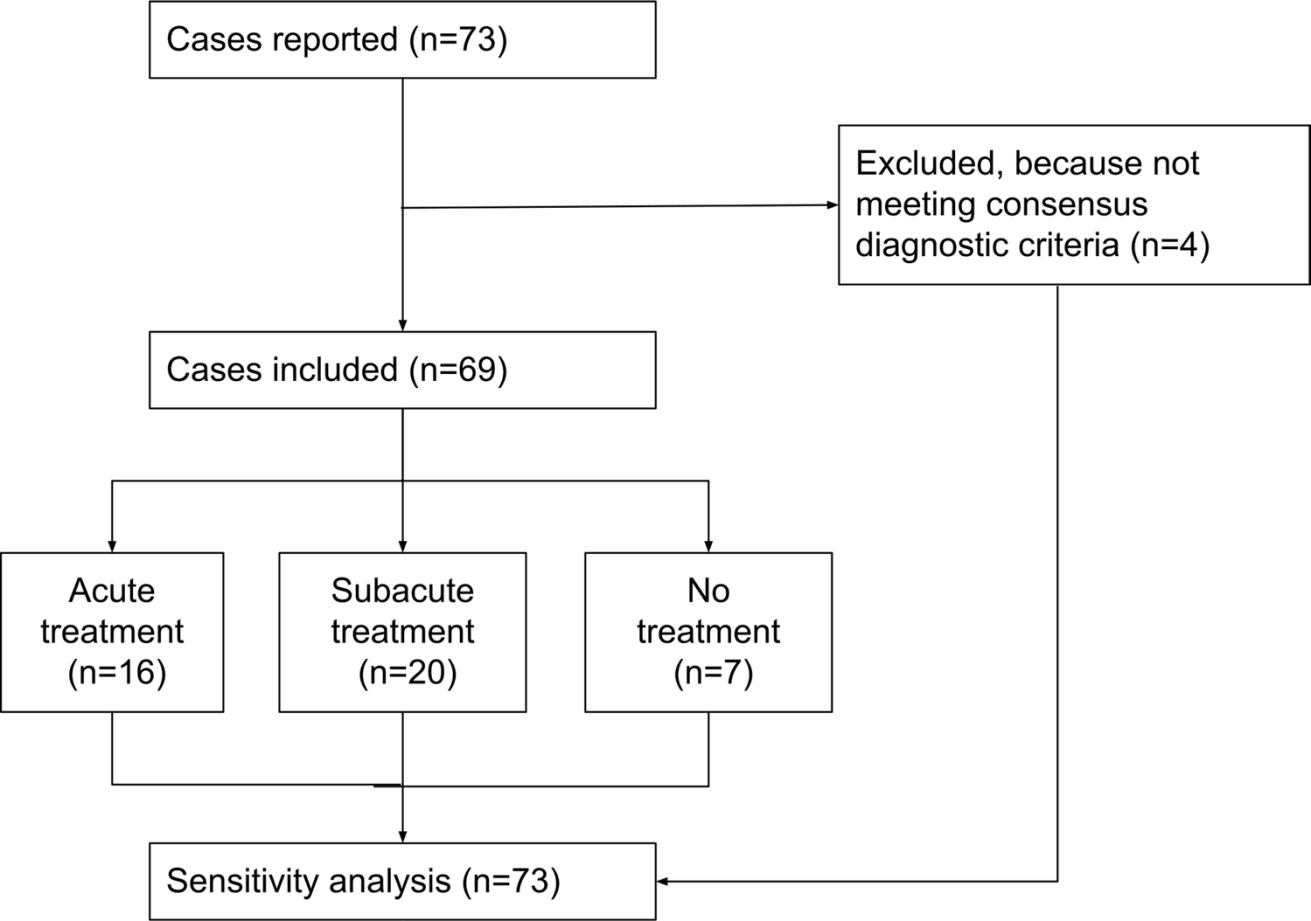
Study flow chart

**Fig. 2:**
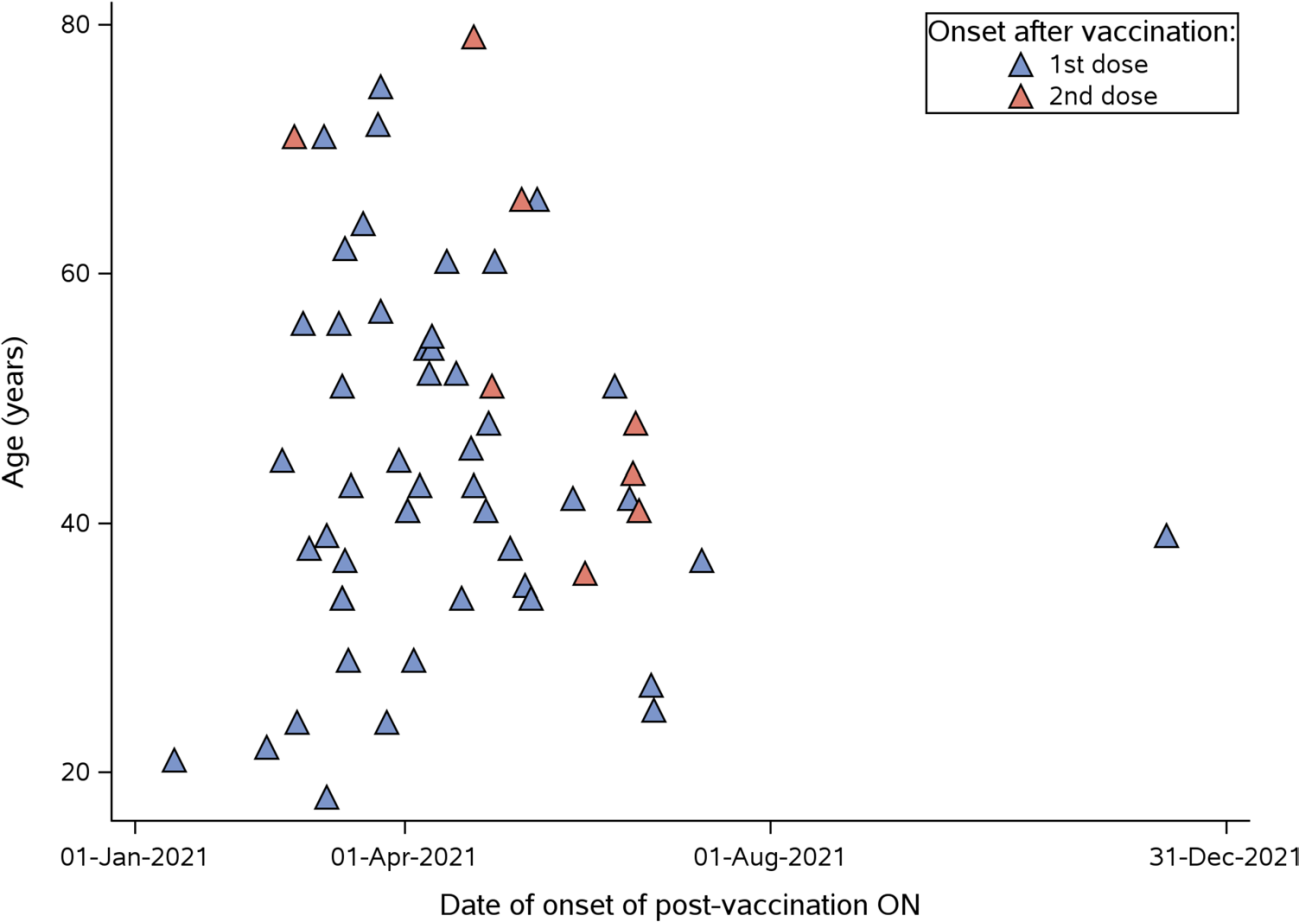
The figure illustrates the global dates of onset of post-vaccination optic neuritis. The graph includes information on whether the onset occurred after the first dose (blue triangle) or the second dose (red triangle) of vaccination. The majority of cases exhibited optic neuritis symptoms following the administration of the first vaccine dose (84%)

Seventy-three individuals were brought to our attention and 69 met the inclusion criteria (Fig. 1). Complete demographics are detailed in Table 1. Most patients were Caucasian (58/69, 84%) and had received the AstraZeneca (AZ) vaccine (43/69, 62%, Table 1). There were no differences between vaccine types for any demographic or clinical variables. In this cohort we had no cases of post-vaccination ON after Moderna, Novavax or Sputnik V, but are aware that this has been reported in the literature [3]. The majority of individuals were of female gender (47/69, 68%) and most episodes of post-vaccination ON occurred after the first dose of vaccination (58/69, 84%). After the second dose of vaccination another 11/69 (16%) individuals developed post-vaccination ON (Fig. 2 and supplementary Fig. 1). Two subjects experienced recurrent post-vaccination ON and one familial association (mother and daughter) was observed. The median time of onset was within 16 days (ranged from 1–69 days) after vaccination, but the majority of events occurred within three weeks (Supplementary Fig. 2). The presentation was unilateral in 52/69 (75%) and bilateral in the remaining 17/69 (25%, Table 1). The majority of individuals (54/69, 78%) reported ocular pain which worsened on eye movements at onset.

**Fig. 3:**
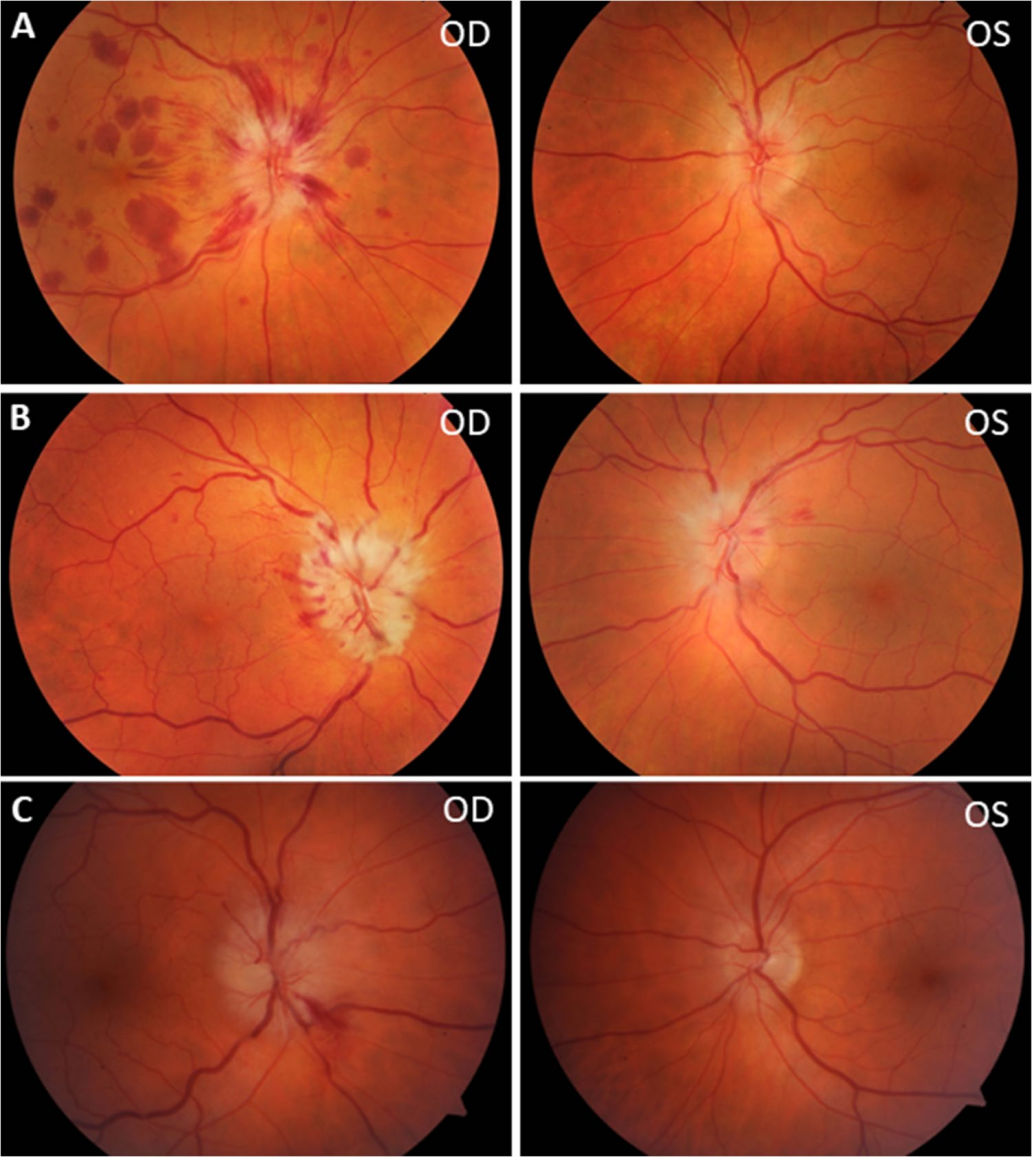
Fundus Color Photographs of Optic Nerve Swelling in Patients with Optic Neuritis and Papillitis. (**A**) Fundus photographs of the right (OD) and left (OS) eyes of the index case in London, a 64-year-old woman with a 48-h history of rapidly progressing bilateral simultaneous visual loss and mild ocular discomfort, occurring two weeks after receiving the AstraZeneca vaccine. The right eye exhibited total visual loss (no light perception), while the left eye had extensive field loss pre-treatment. Notably, there are extensive hemorrhages in the posterior pole, which are unusual in cases of optic neuritis. She was MOG-antibody seronegative. (**B**) Fundus photographs of the right (OD) and left (OS) eyes of a 57-year-old MOG-antibody seropositive patient with bilateral severe asymmetric optic neuritis. The right eye shows marked swelling with peripapillary cotton wool spots and hemorrhages, while the left eye has a lesser degree of swelling. (**C**) Fundus photographs of the right (OD) and left (OS) eyes of a 48-year-old MOG-antibody seropositive patient with bilateral severe asymmetric optic neuritis. Similar to case B, the right eye exhibits significant swelling with peripapillary cotton wool spots and hemorrhages, whereas the left eye shows a lesser degree of swelling

Details of the eye examination, paraclinical tests, past medical history and treatment are summarized in Table 1. For unilateral cases the high contrast visual acuities at presentation and after treatment are shown. For bilateral cases the visual acuity of the worse eye is given. The most common fundus appearance on presentation was papillitis (edema of the optic disc) in 40/69 (58%) which was recorded as severe in 3. In another 3 cases OCT revealed a swollen pRNFL not recognized clinically. Illustrative examples for the acute findings of the optic discs are shown in Fig. 3.

The primary outcome measure, VA, was good at group level (logMAR median 0.0) for post-vaccination ON after any vaccine (Table 1). Four individuals had poor visual outcomes (one logMAR 3.0, one logMAR 1.8, one logMAR 1.2 and two logMAR 0.6). There was a significant correlation between VA at presentation and VA at outcome (R = 0.41, p = 0.0015). There was a weak correlation between time to onset and outcome VA (R = 0.30, p = 0.0185). The outcome VA was not associated with type of vaccine, age, ethnicity, disc appearance on fundoscopy/OCT, presence of ON changes in the MRI or MOG seropositivity (16/69, 23%). Not a single individual was seropositive for AQP4 antibodies (Table 1).

**Fig. 4:**
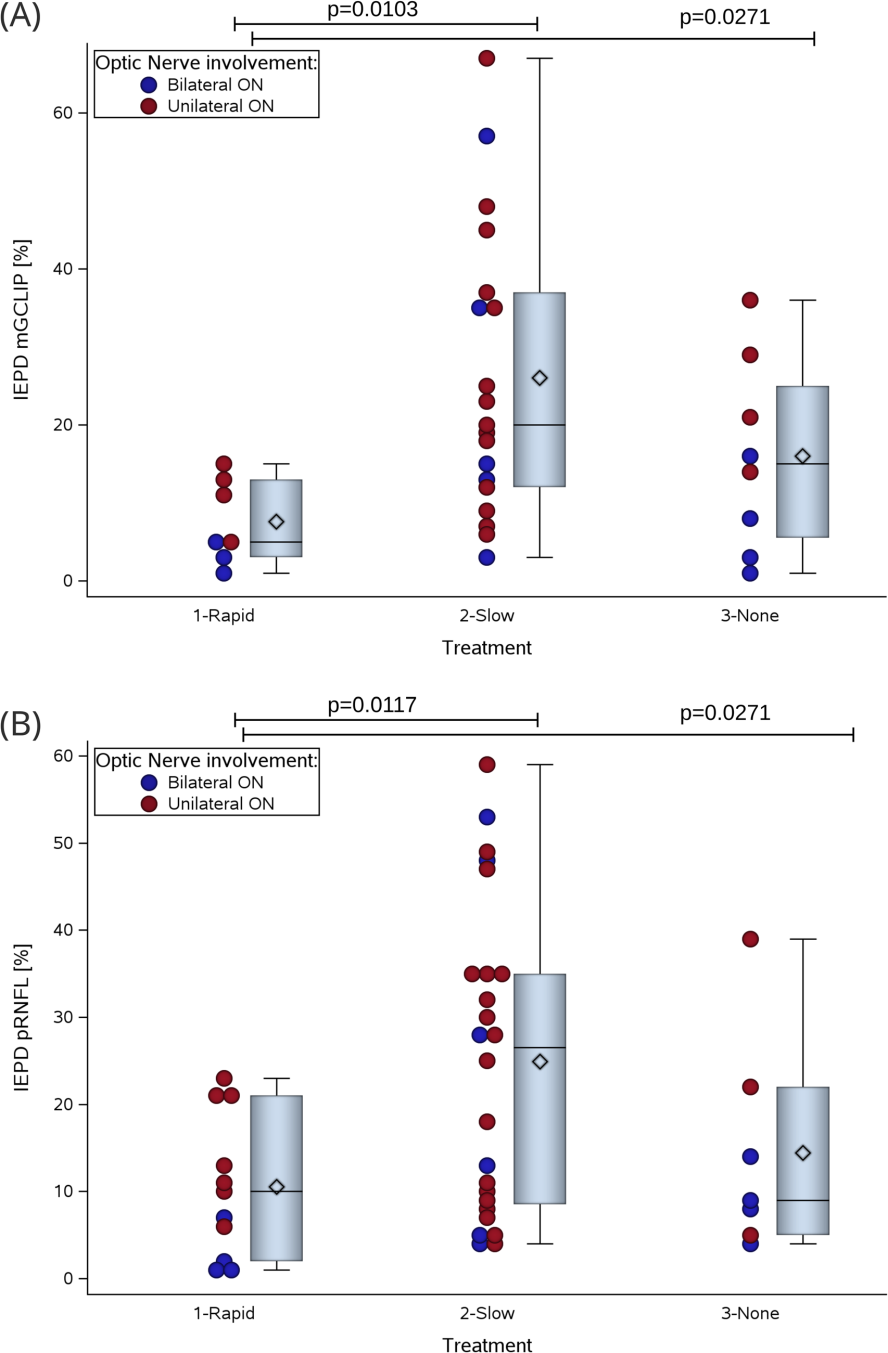
Structural Outcomes of Corticosteroid Treatment in Post-Vaccination Optic Neuritis. (**A**) mGCIPL IEPD and (**B**) pRNFL IEPD outcomes illustrate that corticosteroid treatment administered in the acute phase (0–7 days) results in better structural outcomes compared to delayed treatment in the subacute phase (7 days to 3 months) or no treatment. Patients with post-vaccination optic neuritis are indicated in red for unilateral visual loss and in blue for bilateral visual loss

The secondary outcome measure, the IEPD of the mGCIPL, correlated significantly with time to treatment with corticosteroids (R = 0.63, p = 0.0005). There was no correlation of the mGCIPL IEPD with age (R = 0.079, p = 0.65), time to onset (R = −0.013, p = 0.94), VA at onset (R = 0.174, p = 0.33) or time to analgesia after start of treatment (R = 0.015, p = 0.95). The significance of the finding of a correlation between the size of the mGCIPL IEPD and time to onset of treatment was preserved in a subgroup analysis of individuals with unilateral onset (R = 0.51, p = 0.03), but was narrowly lost for the few patients (Table 1) with bilateral onset (R = 0.67, p = 0.06). Early corticosteroid treatment of ON (0–7 days from onset of first symptom) was associated with a better secondary outcome (less atrophy) of the IEPD mGCIPL (p = 0.0271) on a group level (Fig. 4 A) when compared to delayed start of treatment with corticosteroids in the subacute phase (7 days to 3 months from onset of first symptom) or no treatment. The effect retained significance with a post-hoc analysis comparing treatment in the acute with the subacute phase of ON (p = 0.0103).

In a subgroup analysis we also compared the pRNFL IEPD between treatment groups (Fig. 4 B). Overall the outcome pRNFL IEPD correlated significantly with time to start of treatment (R = 0.50, p = 0.0021). On a group level there was a significant difference for the pRNFL IEPD (p = 0.0271) between individuals treated in the acute phase compared to the subacute phase or untreated individuals. Again, the post-hoc analysis showed less severe atrophy in individuals treated acutely compared to those treated in the subacute phase of ON (p = 0.0117).

The UK was the first nation to implement population-level vaccination (1st dose of vaccine given, outside a clinical trial, to Margaret Keenan on 08-DEC-2020) and, within this cohort, it has the highest number of post-vaccination optic neuritis cases (n = 31). There was an association in time between the age groups vaccinated and occurrence of post-vaccination ON in the UK (Supplementary Fig. 1). There was a trend for younger individuals to present at a later date with post-vaccination ON compared with older age groups who received their vaccinations earlier in the UK. The shift from younger to older age groups is shown by comparison with recent pre-pandemic epidemiological data (Supplementary Fig. 3).

### Radiological findings

MRI brain and orbit scans for 24 UK patients were scored and details can be seen in supplementary tables 2–4. Gadolinium was given in 21 of these cases. The coronal and axial sections of typical MRI examples are shown in Supplementary Fig. 4. Evidence of optic nerve segmental T2/STIR hyper-intensity was seen in n = 23 patients: 8 bilateral, 15 unilateral, 1 negative. Of these 23 abnormal scans, associated gadolinium enhancement was seen in 16/20 patients, and optic nerve swelling in 17/23 (see columns 3,6 and 7 in supplementary Table 2). Out of the four gadolinium-negative patients, two had optic nerve swelling and two showed T2 optic nerve hyper-intensity without enhancement or swelling (patients 2,14). All three patients who did not receive gadolinium had segmental optic nerve swelling (patients 11, 13 and 19 in supplementary Table 2). Five of the 23 abnormal scans demonstrated multiple white matter lesions consistent with inflammatory demyelination (four unilateral ON, one bilateral ON). Two other patients had single acute neuro-inflammatory lesions (1 midbrain, 1 temporal) in addition to bilateral ON.

Optic nerve sheath enhancement was noted in four of 13 abnormal right and seven of 16 abnormal left optic nerves given contrast. It was present in three out of the eight bilateral cases given contrast. For unilateral cases, mean STIR lesion length was 20.88 mm (median 20.80, range 4–40) and mean gadolinium lesion length was 21.08 mm (median 18.9, range 4–40). For bilateral cases, mean STIR lesion length was 15.89 mm (median 12, range 4–42.9) and mean gadolinium lesion length was 20.46 mm (median 22.89, range 6–36).

### Sensitivity analyses

Sensitivity analyses were conducted to assess the robustness of the findings from the primary analysis of the outcome variables. Specifically, the sensitivity analyses evaluated whether excluding the four outlier patients (Fig. 1), who had longer time intervals from vaccination to symptom onset, would have changed the results. The significance of the correlation between VA at presentation and VA at outcome remained robust in the sensitivity analysis (R = 0.41, p = 0.0015), as was the correlation between time to onset and outcome VA (R = 0.30, p = 0.0185). Additionally, the correlation between time to treatment and the mGCIPL IEPD (R = 0.62, p = 0.0004) and pRNFL IEPD (R = 0.395, p = 0.0141) was also upheld.

Furthermore, the sensitivity analysis provided further support for the statistically significant findings regarding the effect of corticosteroid treatment in the acute phase compared to delayed or no treatment (see also supplemenatry Table 4). Specifically, there was a significant difference observed for the mGCIPL IEPD (p = 0.019) at the group level, and in the post-hoc analysis comparing treatment in the acute phase with the subacute phase of ON (p = 0.0074). Although the statistical significance for the pRNFL IEPD was narrowly missed at the group level in the sensitivity analysis (p = 0.0527), it remained significant in the post-hoc analysis (p = 0.0276).

## Discussion

Post-COVID-19 vaccination ON is a rare complication with only anecdotal reports in the literature. For timely comparison with recent small pox vaccination efforts against the emerging monkeypox virus, a large retrospective US Veterans study identified two patients with post-vaccination ON after small pox, but did not describe the clinical phenotype or treatment response [15]. Present prospective study with 69 deep phenotyped patients meeting consensus diagnostic criteria adds valuable information on patient management. The unique timing of the SARS-CoV-2 pandemic which coincided with the establishment of the ICON 2022 consensus diagnostic criteria for ON and the mandatory roll out of vaccination has made it possible to study, a group of individuals presenting with post-vaccination ON. The group is homogeneous with regard to meeting diagnostic criteria for definite ON [4]. Here we report the detailed clinical and paraclinical follow-up in 69 individuals presenting with post-vaccination ON previously only shared in form of a pre-print [1]. The final biomarker based subgroup classification of all cases heterogeneity ranging from an isolated post-vaccination ON, over demyelinating disease to MOG autoimmunity. However, heterogeneity of subgroups aside, the main finding is that visual outcome is excellent in almost all patients during the one year follow-up period. Rapid treatment with corticosteroids in the acute phase (< 7 days after onset of symtoms) of post-vaccination ON, rather than delayed treatment or no treatment, also improves structural outcome with less severe atrophy of the mGCIPL and pRNFL.

The clinical picture of severe post-vaccination ON with pain worsening on eye movements (78%), frequent bilateral involvement, MOG-IgG seropositivity, significant papillitis or extensive length of optic nerve lesions strongly suggests a systemic autoimmune process in many of these patients.

Significantly, within our post-vaccination ON cohort, we identified 15 individuals who tested positive for MOG antibodies. Several of these cases exhibited clinical and radiological features consistent with MOG antibody-associated disorder (MOGAD) [16], reinforcing our initial data shared only as a preprint [1] Subsequent reports have further emphasized the link between MOG seropositivity and SARS-CoV-2 vaccination [17, 18] Indeed there are data supporting the occurrence of cross-reactivity between SARS-CoV-2 vaccination and IgG antibodies against MOG [19]. Importantly for clinical management, MOG-ON is an extremely steroid responsive condition requiring early treatment to protect vision and retinal neurons [20–22]. The findings of these independent studies align with our study, which also introduces OCT IEPD as a valuable, device independent, structural outcome measure.

Regarding the treatment, there is emerging agreement in the literature on acute treatment with high-dose methylprednisolone, PLEX or IVIG [5, 23–25], and most reports of post-vaccination and post-infectious ON have been treated with corticosteroids, since an immune-mediated mechanism is also presumed [26]. In our study acute ON was defined as occurring within 0–7 days [4] which aligns with the average corticosteroid initiation time in the ON Treatment trial [27]. Twenty patients were treated within this time frame and recovery of high contrast VA was excellent. All four patients with a poor visual outcome had their high dose corticosteroids more than 10 days after onset of symptoms. We accept, as a study limitation, that our primary outcome measure, high contrast VA, whilst most commonly used, remains a relatively crude functional parameter of optic nerve recovery. Therefore it is important to note that also our secondary outcome measure, the mGCIPL IEPD, did show a better outcome with early corticosteroid treatment. This result is robust because the OCT findings retained their level of significance in a subgroup analysis for the pRNFL IEPD and sensitivity analyses.

Geographical mapping of the ON incidence during the study period showed the highest incidence in the UK permitting for a detailed analysis in relationship to national vaccine roll-out. This revealed a close age group related association between date of vaccination and onset of ON (supplementary Fig. 3). In our study, we found a much higher proportion of AstraZeneca than Pfizer vaccinations, and some cases following Sinovac. This is consistent with the findings of national UK database study [2]. Interestingly, despite increasing awareness in the UK neuro-ophthalmology community, the numbers of post-vaccination ON seem to have peaked between March and April 2021 after a priming dose. In a smaller proportion post-vaccination ON was observed after the second dose, which was in all cases with the same type of vaccine. Combined, this suggest that the incidence of post-vaccination ON may be highest with the first injection of a novel vaccine.

As another limitation to our study, we acknowledge the lack of solid epidemiological data on the incidence and prevalence of ON in the population with and without vaccination, which currently precludes calculation of odds ratios (OR). From present data, however, there is no evidence to suggest that the incidence of ON has increased in the UK due to vaccination [28]. Only future re-analysis of the UK epidemiological data will reveal if there has been a change of incidence during the pandemic if compared to the historical data. Therefore another limitation is the lack of a prospective, time matched, control group. This was not possible due to the restricted access to clinical services during lock-down, affecting some countries more than others. Likewise we have not identified any pediatric cases because in some countries there is a medicolegal age restriction for services which could have introduced a bias.

A further limitation is that three different OCT devices were used in this study. To overcome this, we have reported percentage measures of retinal asymmetry which are understood to be device independent [4]. We can therefore only indirectly report on the degree of atrophy by comparing the mGCIPL thickness between both eyes at onset and last follow-up, where an increase of the mGCIPL IEPD indicates an increase of asymmetric atrophy [4]. An analytical limitation of the MOG auto-antibody tests is the variability in assay methodologies used worldwide. Not all samples were assessed using the recommended cell-based assays, which offer superior analytical sensitivity [31]. Consequently, the actual number of post-vaccination MOG-ON cases may be underestimated. Additionally, longitudinal testing of MOG auto-antibodies would have been beneficial in distinguishing cases with a transient post-vaccination autoimmune response from those at risk of developing a relapsing disease course. Accepting this limitation, there is no evidence for vaccination to cause harm in subjects with known MOGAD [32].

In conclusion, our study reveals the presence of a distinct, albeit relatively small, epidemic cluster of post-vaccination ON, primarily observed in the UK, which shares similar clinical characteristics with cases reported worldwide. Notably, this observation was made for three of the vaccines on the market with no statistically significant difference in presenting severity, visual outcome, or steroid responsiveness. However, our findings underscore the relevance of MOG antibody testing in post-vaccination ON [19]. The data also highlights the importance of timely intervention during the acute phase, as delays in treatment were associated with more pronounced retinal atrophy on optical coherence tomography. It is crucial to inform individuals which undergo vaccination about the rare possibility of developing post-vaccination ON. Physicians and patients need to know that functional visual recovery can be expected to be excellent. Treatment in the acute phase may further improve the structural outcome.

## Electronic supplementary material

Below is the link to the electronic supplementary material.

ESM 1 The dot plot illustrates the relationship between the age of the study group, the rollout dates of the vaccines, and the onset of optic neuritis (ON) in 30 individuals from the UK. The red vertical reference lines indicate the dates of vaccine rollout for specific age groups. The dots represent the dates of optic neuritis onset. In March 2021, we identified a unique cluster of optic neuritis cases among older patients in Birmingham and London following vaccination. These cases presented as either severe unilateral optic neuritis (coloured in red) or bilateral optic neuritis (coloured in blue). Bilateral simultaneous optic neuritis, regardless of additional clinical details, strongly suggests a systemic cause (PNG 105 KB)

Supplementary file1 (TIFF 8789 KB)

ESM 2 The histogram depicts the time interval in days from vaccination to the onset of post-vaccination optic neuritis in the 69 individuals who fulfilled the ICON 2022 diagnostic criteria (PNG 94.1 KB)

Supplementary file2 (TIFF 8789 KB)

ESM 3 The Figure illustrates the age distribution of individuals with post-vaccination optic neuritis in 2021 (blue line) compared to historical data from the UK (1997-2018) on optic neuritis attributed to other causes (red dashed line). A noticeable shift from a younger age group to an older age group is observed in the incidence of post-vaccination optic neuritis (PNG 137 KB)

Supplementary file3 (TIFF 8789 KB)

ESM 4 Examples of Magnetic Resonance Imaging (MRI) scans in post-vaccine optic neuritis cases from our study illustrating a pattern of radiological findings commonly found in immune-mediated optic neuritis. MRI scans shown for both MOG seropositive and seronegative patients. A,C,D,E. All images are coronal post-Gd, fat-suppressed, T1 orbital or intracranial views. A. Prominent bilateral intraorbital optic nerve enhancement (MOG seronegative). B. View of right optic nerve enhancement in the immediate retrobulbar portion (fat saturation artefact left orbit) in an MOG seronegative patient with bilateral optic neuritis. C. Left anterior optic nerve and peri-neural sheath enhancement in an Ig-G MOG positive patient. D. Left superiortemporal gyrus and left middle temporal gyrus ring-enhancing lesions in a patient presenting with simultaneous bilateral optic neuritis (MOG seronegative). E. Posterior enhancement of the intracranial portion of the right optic nerve (MOG seronegative). F and G correspond to post-gad fat-suppressed T1 post-contrast MRI scan axial views. F. Enhancement of the whole left optic nerve from retrobulbar to intracranial portions in an MOG seropositive patient. G. Bilateral enhancement of both optic nerves in the intraorbital portion in an MOG seronegative patient. H. Axial T2 weighted STIR views show hyperintensity affecting the left optic nerve in its whole length (MOG seronegative) (PNG 2.41 MB)

Supplementary file4 (TIFF 5692 KB)

ESM 5 Familial cases, the 64-Year-Old Mother presented with ocular pain which was followed by subacute loss of vision in the right eye down to perception of light and in the left eye 6/9.5 (high contrast Snellen acuity) with bilateral dyschromatopsia and a right RAPD. Her COVID-19 tests were negative and remained negative on serial follow-up. She had received the AZ vaccine against COVID-19 14 days prior to onset of her eye symptoms. (A) At baseline, bilateral optic disc swelling with disc hemorrhages is observed (OD, Topcon Triton-1000). The hemorrhages extend into the macula. The red-free image enhances the visualization of the hemorrhages’ distribution in the retina. The corresponding OCT, taken along the horizontal green line in the fundus photograph and the red-free image, reveals retinal thickening in the outer temporal and inferior sectors of the EDTRS grid. (B) At one-year follow-up, the disc swelling has resolved, and the hemorrhages have been absorbed. The OCT now shows atrophy in all sectors of the EDTRS grid except the outer nasal area. (C) OCT of the swollen right disc at presentation, and (D) OCT of the swollen left disc. Both OCTs are from the same time point as the image shown in A. (E) A repeat quantitative assessment of pRNFL thickness with the Heidelberg Spectralis OCT indicates that the degree of pRNFL atrophy remains stable between the 6-month and 12-month follow-ups. (F) Similarly, pRNFL atrophy remains stable in the left eye (LE). Her final high-contrast visual acuities were RE 6/9, Ishihara 15/17, and LE 6/7.5, Ishihara 17/17. Her Daughter also experienced pain worsening on eye movements two weeks after receiving the Pfizer vaccine against COVID-19. The daughter recognised this as the initial symptom of post-vaccination optic neuritis from her mother's history and immediately presented to the hospital where a swollen optic disc was seen and high dose treatment with corticosteroids was initiated instantaneously. Within hours from starting the corticosteroid infusion the pain stopped, disc swelling had resolved at follow-up and visual loss was prevented altogether (PNG 1.96 MB)

Supplementary file5 (TIFF 11812 KB)

Supplementary file6 (DOCX 60 KB)

## Appendix 1

**Table:**

First Name	Second Name
Ahmed	Abdel-Hay *
Dália	Meira *
Daniah	Alshowaeir *
Michael	Ashenhurst *
Mashair	Bakheet *
Oscar	Balaguer *
Ruchika	Batra *
Zelie	Britton *
Wallace	Brownlee *
Ailbhe	Burke *
Anna	Camós-Carreras *
Fiona	Costello *
Sarah	Coulette *
Nieves	De Las Rivas Ramirez *
Romain	Deschamps *
Jose	Flores-Rivera *
Clare L.	Fraser *
Caroline	Froment Tilikete *
Lorna	Galleguillos*
José Manuel	Guajardo *
Héctor F	Jiménez-Ortiz *
Lisa	Lagrou *
Pierre	Lardeux *
Ruth	Martín *
Katherine	Miszkiel *
Silvia	Muñoz *
Lola	Ogunbowale *
Eoin	O’Sullivan *
Chris	Ovens *
Friedemann	Paul *
Alfonso	Rodriguez-Morales
Federico	Sadun *
Bernardo	Sanchez-Dalmau *
Aksel	Siva *
Muriel	Spöeri *
Kurt-Wolfram	Sühs *
Anand	Trip *
Melih	Tutuncu *
Mike P	Wattjes *
John	Woolmore *
Jasmin	Zvornicanin *
