# Correction: Treatment of post-vaccination optic neuritis: implications from the global SARS-CoV-2 vaccination effort

**DOI:** 10.1007/s00417-026-07129-z

**Published:** 2026-01-29

**Authors:** Yan Ning Neo, Lidia Martinez-Alvarez, Indran Davagnanam, Gabriela Girafa, Fion Bremner, Tasanee Braithwaite, Zhaleh Khaleeli, Victoria Nowak, Ahmed T. Toosy, Axel Petzold

**Affiliations:** 1 Moorfields Eye Hospital NHS Foundation Trust, 162 City Road, London, EC1V 2PD UK; 2 Birmingham and Midland Eye Centre, Sandwell and West, Birmingham Hospitals NHS Trust, Birmingham, UK; 3 Queen Elizabeth Hospital Birmingham, UniversityHospitalsBirmingham, Birmingham, UK; 4 National Hospital for Neurology and Neurosurgery, University College London Hospitals NHS Foundation Trust, London, Queen Square UK; 5 King’s Health Partners Centre for Translational Medicine, London, UK; 6 Guy’s and St Thomas’ NHS Foundation Trust, London, UK; 7 Dept. Of Neuroinflammation, Queen Square MS CentreUCLSquare Institute of NeurologyUniversity College London, London, UK

**Graefe's Archive for Clinical and Experimental Ophthalmology**

**https://doi.org/10.1007/s00417-025–06805-w**.

In the online version of this article, there has been a correction regarding the author list under the institutional group "International Consortium on Optic Neuritis (ICON)." to be internally consistent with the names listed in Appendix 1 of the article. Specifically, James Acheson has since retired and asked to be removed, Dália Meira has been added and Chris Ovens was misspelled as Owens.

The original article has been corrected.

