# Supplementary figures and images for: Treatment of post-vaccination optic neuritis: implications from the global SARS-CoV-2 vaccination effort
Source: Graefes Arch Clin Exp Ophthalmol. 2025 Nov 29;264(3):851–62. doi: 10.1007/s00417-025-06805-w (PMC12966182; doi:10.1007/s00417-025-06805-w)

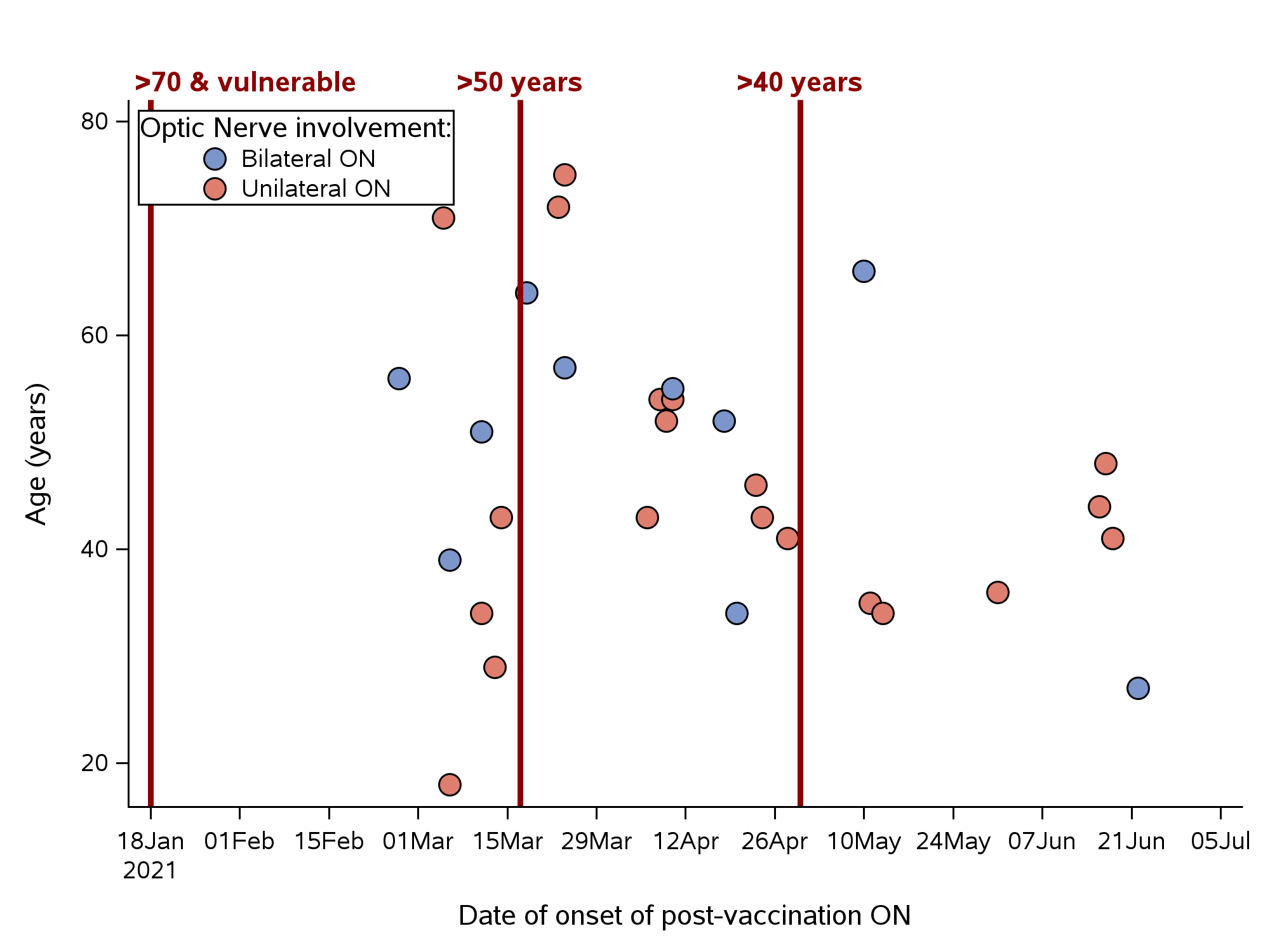

Supplement: Supplementary file 1 — The dot plot illustrates the relationship between the age of the study group, the rollout dates of the vaccines, and the onset of optic neuritis (ON) in 30 individuals from the UK. The red vertical reference lines indicate the dates of vaccine rollout for specific age groups. The dots represent the dates of optic neuritis onset. In March 2021, we identified a unique cluster of optic neuritis cases among older patients in Birmingham and London following vaccination. These cases presented as either severe unilateral optic neuritis (coloured in red) or bilateral optic neuritis (coloured in blue). Bilateral simultaneous optic neuritis, regardless of additional clinical details, strongly suggests a systemic cause (PNG 105 KB) [file 417_2025_6805_Fig5_ESM.png]

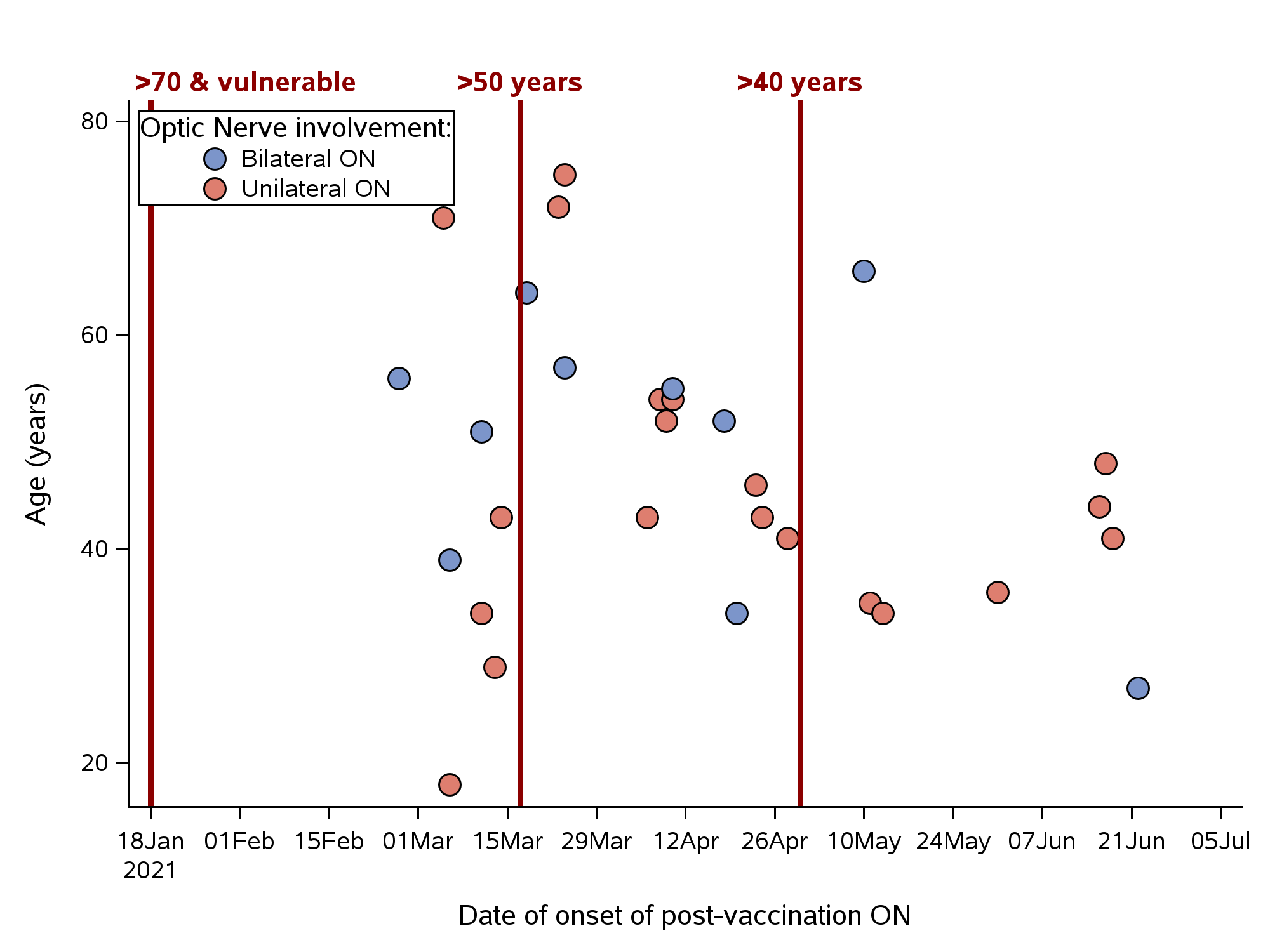

Supplement: Supplementary file 2 — Supplementary file1 (TIFF 8789 KB) [file 417_2025_6805_MOESM1_ESM.tiff]

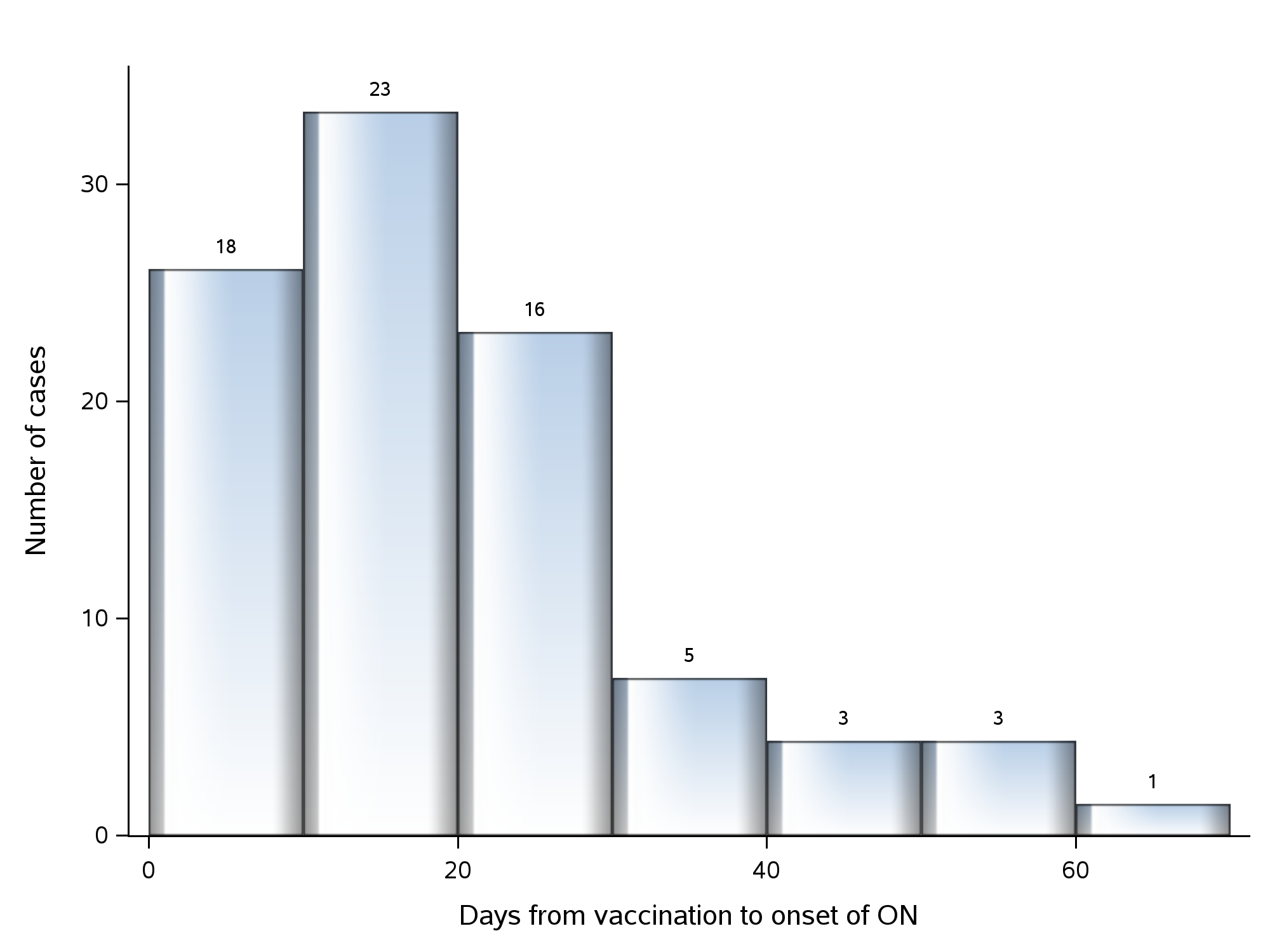

Supplement: Supplementary file 3 — The histogram depicts the time interval in days from vaccination to the onset of post-vaccination optic neuritis in the 69 individuals who fulfilled the ICON 2022 diagnostic criteria (PNG 94.1 KB) [file 417_2025_6805_Fig6_ESM.png]

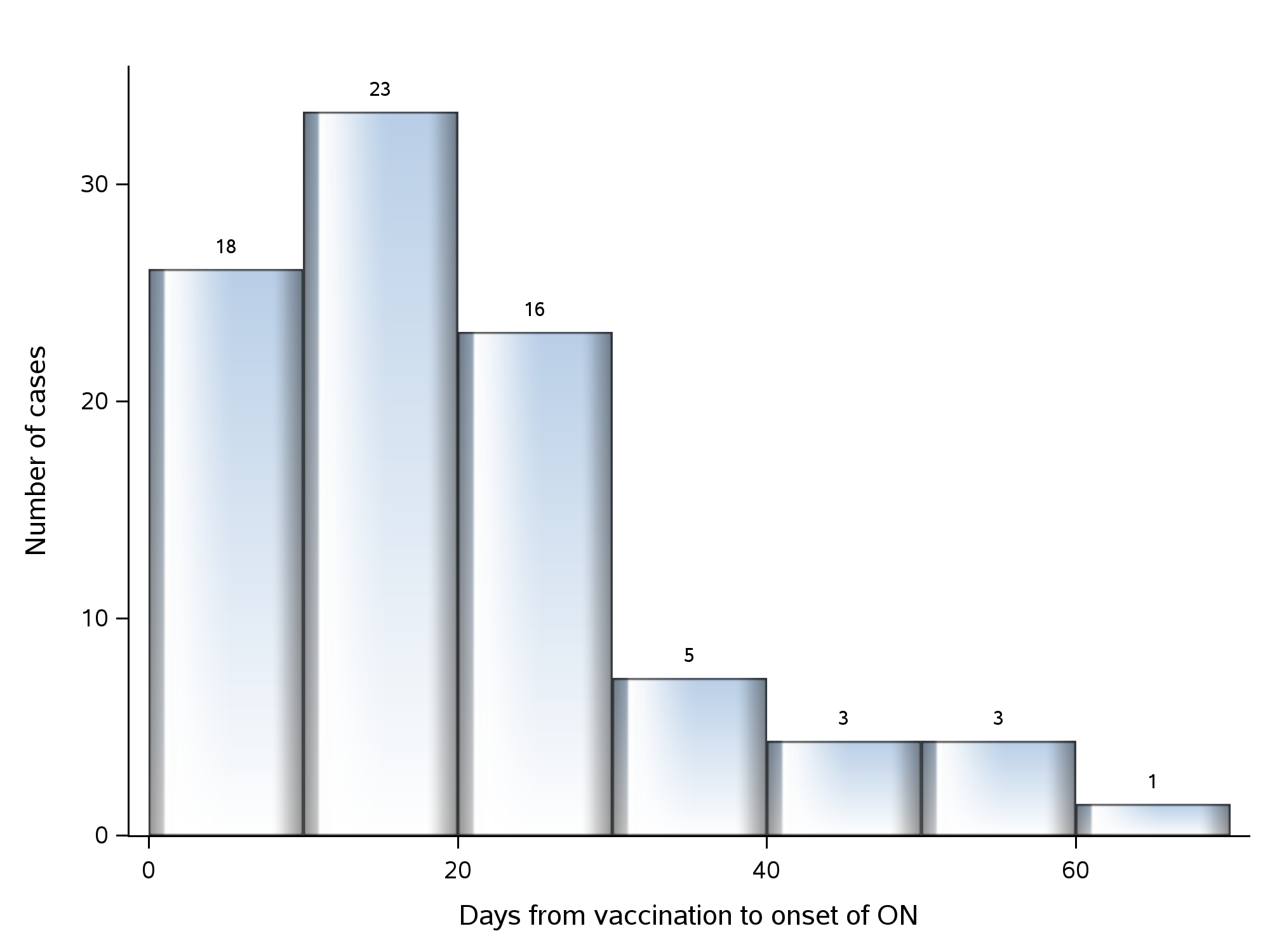

Supplement: Supplementary file 4 — Supplementary file2 (TIFF 8789 KB) [file 417_2025_6805_MOESM2_ESM.tiff]

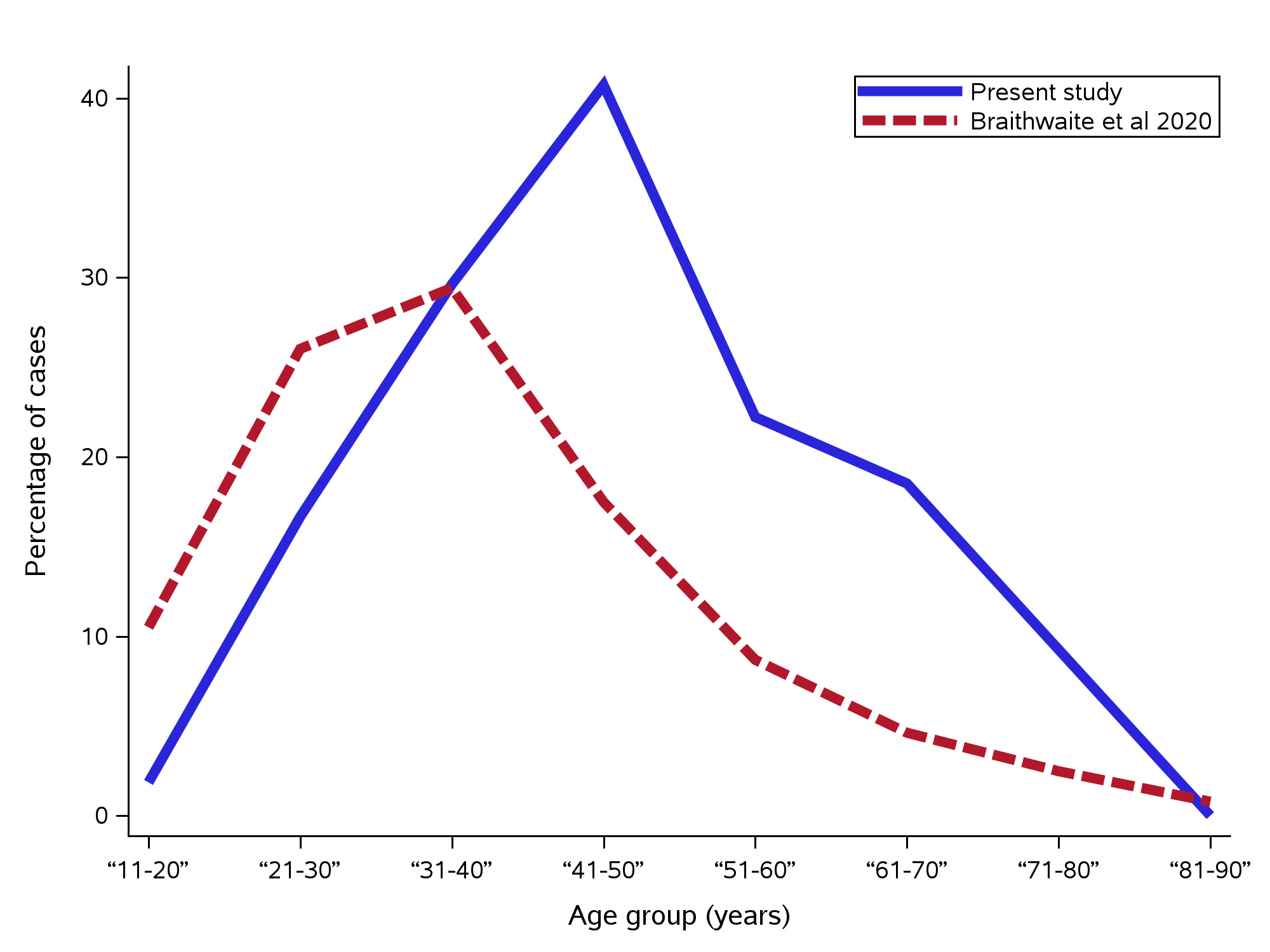

Supplement: Supplementary file 5 — The Figure illustrates the age distribution of individuals with post-vaccination optic neuritis in 2021 (blue line) compared to historical data from the UK (1997-2018) on optic neuritis attributed to other causes (red dashed line). A noticeable shift from a younger age group to an older age group is observed in the incidence of post-vaccination optic neuritis (PNG 137 KB) [file 417_2025_6805_Fig7_ESM.png]

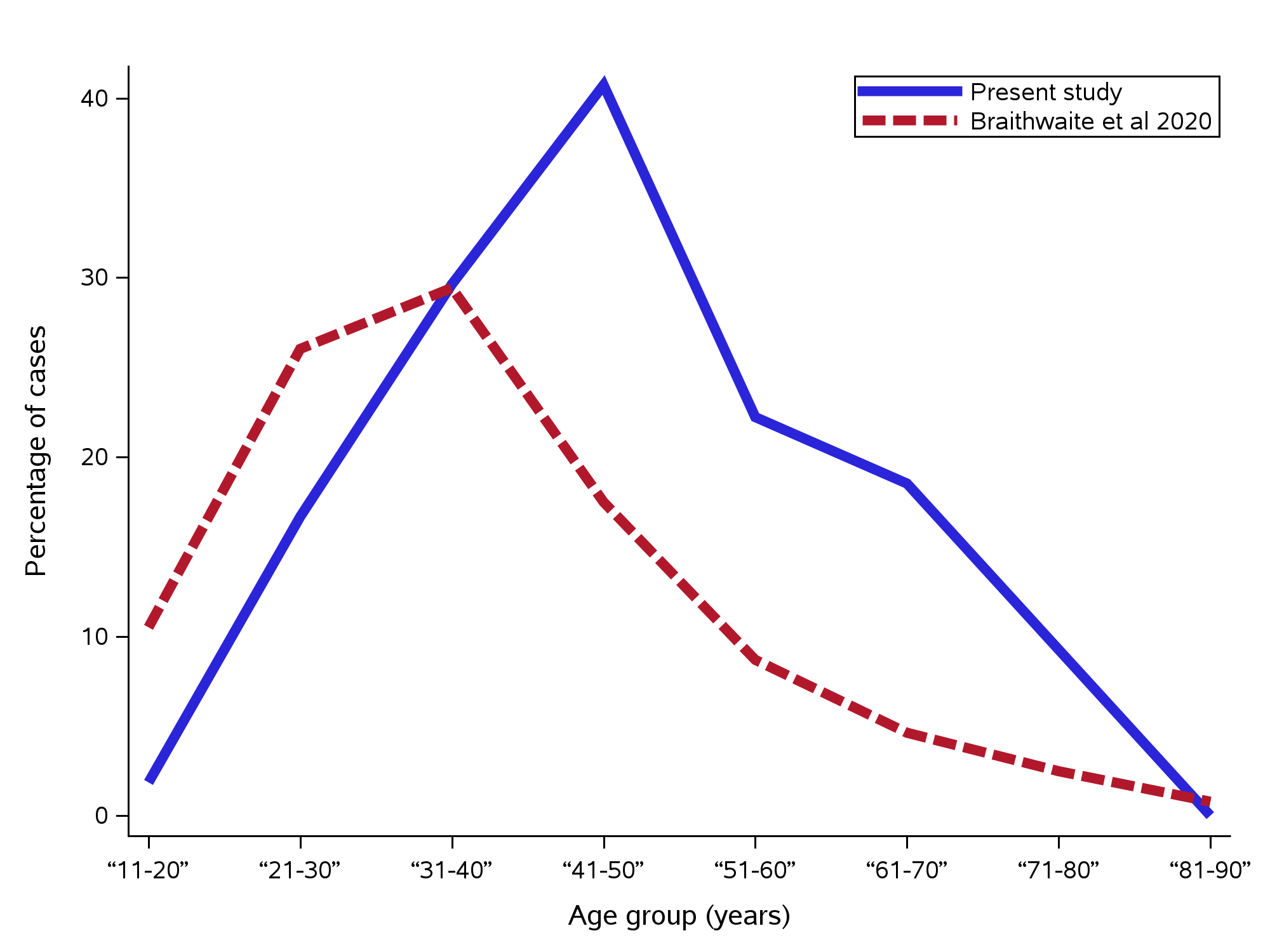

Supplement: Supplementary file 6 — Supplementary file3 (TIFF 8789 KB) [file 417_2025_6805_MOESM3_ESM.tiff]

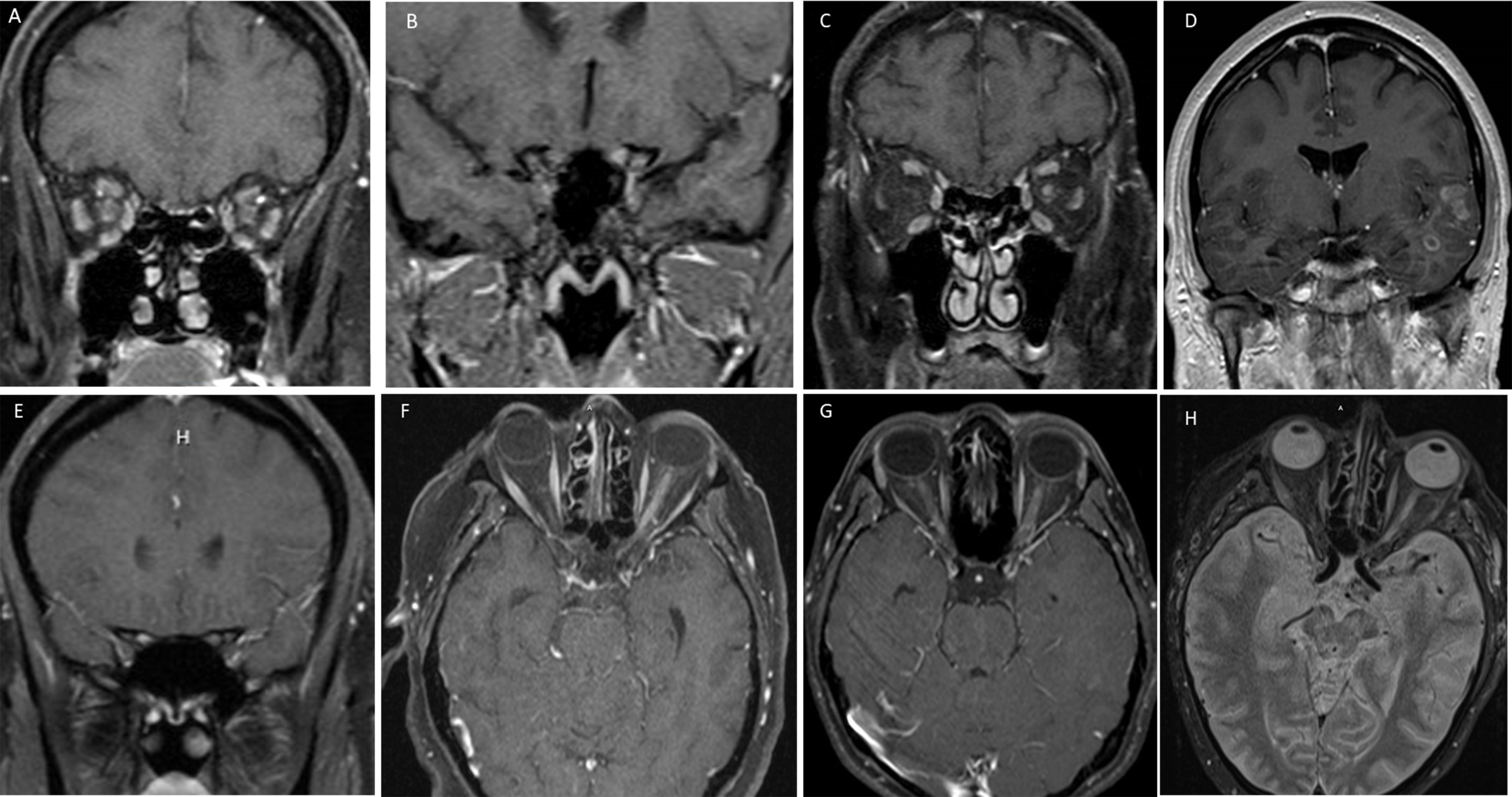

Supplement: Supplementary file 7 — Examples of Magnetic Resonance Imaging (MRI) scans in post-vaccine optic neuritis cases from our study illustrating a pattern of radiological findings commonly found in immune-mediated optic neuritis. MRI scans shown for both MOG seropositive and seronegative patients. A,C,D,E. All images are coronal post-Gd, fat-suppressed, T1 orbital or intracranial views. A. Prominent bilateral intraorbital optic nerve enhancement (MOG seronegative). B. View of right optic nerve enhancement in the immediate retrobulbar portion (fat saturation artefact left orbit) in an MOG seronegative patient with bilateral optic neuritis. C. Left anterior optic nerve and peri-neural sheath enhancement in an Ig-G MOG positive patient. D. Left superiortemporal gyrus and left middle temporal gyrus ring-enhancing lesions in a patient presenting with simultaneous bilateral optic neuritis (MOG seronegative). E. Posterior enhancement of the intracranial portion of the right optic nerve (MOG seronegative). F and G correspond to post-gad fat-suppressed T1 post-contrast MRI scan axial views. F. Enhancement of the whole left optic nerve from retrobulbar to intracranial portions in an MOG seropositive patient. G. Bilateral enhancement of both optic nerves in the intraorbital portion in an MOG seronegative patient. H. Axial T2 weighted STIR views show hyperintensity affecting the left optic nerve in its whole length (MOG seronegative) (PNG 2.41 MB) [file 417_2025_6805_Fig8_ESM.png]

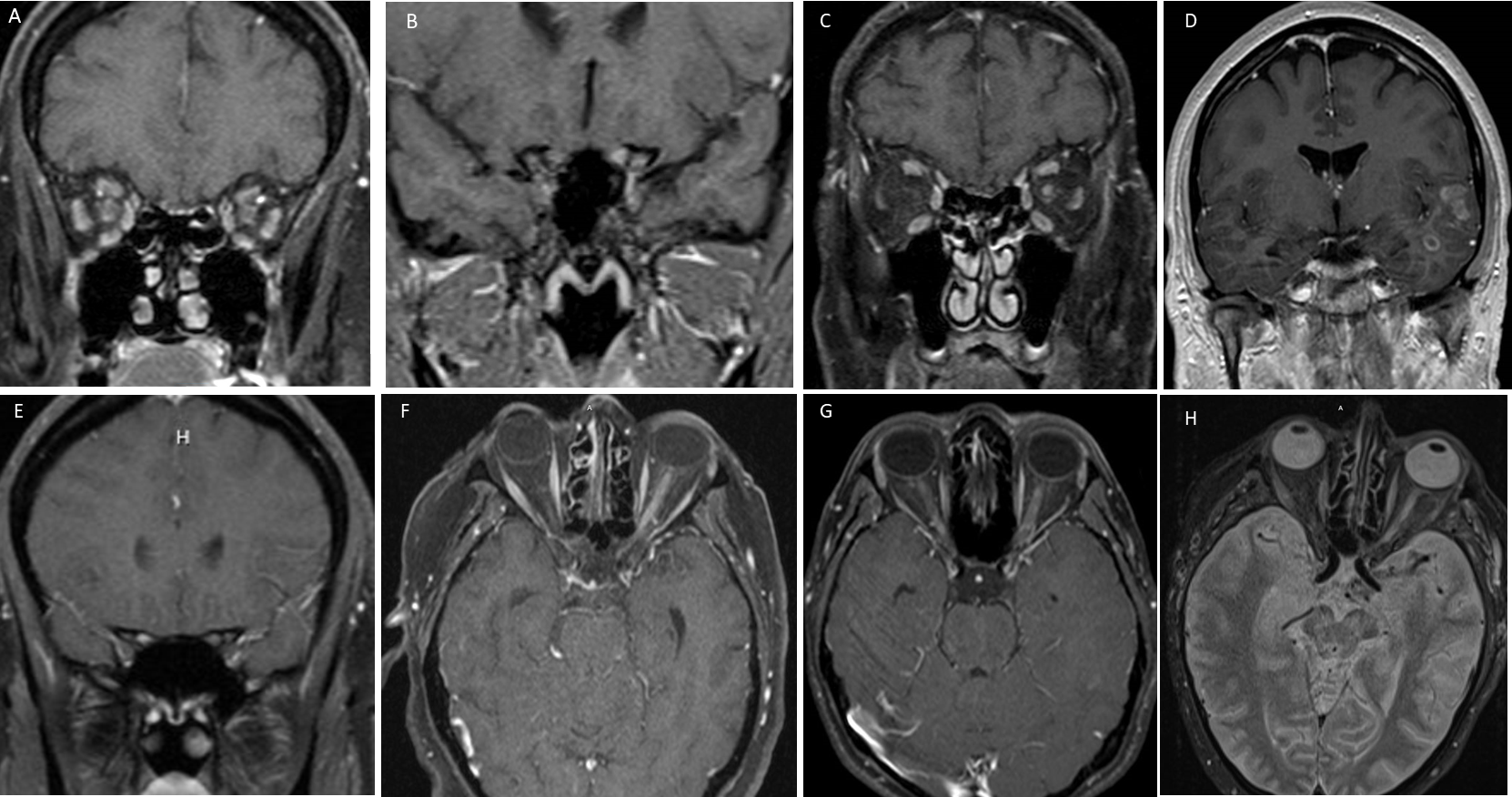

Supplement: Supplementary file 8 — Supplementary file4 (TIFF 5692 KB) [file 417_2025_6805_MOESM4_ESM.tiff]

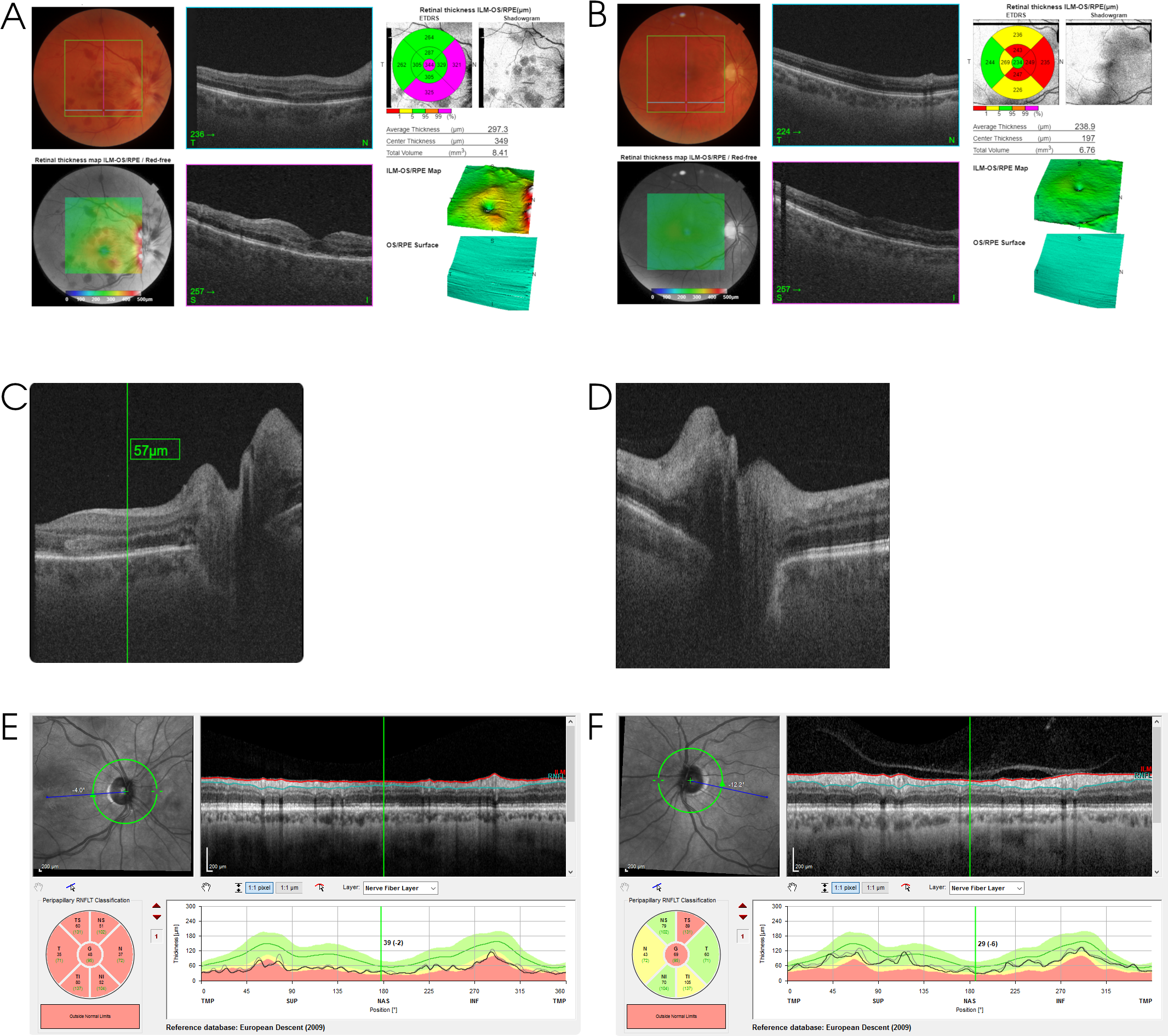

Supplement: Supplementary file 9 — Familial cases, the 64-Year-Old Mother presented with ocular pain which was followed by subacute loss of vision in the right eye down to perception of light and in the left eye 6/9.5 (high contrast Snellen acuity) with bilateral dyschromatopsia and a right RAPD. Her COVID-19 tests were negative and remained negative on serial follow-up. She had received the AZ vaccine against COVID-19 14 days prior to onset of her eye symptoms. (A) At baseline, bilateral optic disc swelling with disc hemorrhages is observed (OD, Topcon Triton-1000). The hemorrhages extend into the macula. The red-free image enhances the visualization of the hemorrhages’ distribution in the retina. The corresponding OCT, taken along the horizontal green line in the fundus photograph and the red-free image, reveals retinal thickening in the outer temporal and inferior sectors of the EDTRS grid. (B) At one-year follow-up, the disc swelling has resolved, and the hemorrhages have been absorbed. The OCT now shows atrophy in all sectors of the EDTRS grid except the outer nasal area. (C) OCT of the swollen right disc at presentation, and (D) OCT of the swollen left disc. Both OCTs are from the same time point as the image shown in A. (E) A repeat quantitative assessment of pRNFL thickness with the Heidelberg Spectralis OCT indicates that the degree of pRNFL atrophy remains stable between the 6-month and 12-month follow-ups. (F) Similarly, pRNFL atrophy remains stable in the left eye (LE). Her final high-contrast visual acuities were RE 6/9, Ishihara 15/17, and LE 6/7.5, Ishihara 17/17. Her Daughter also experienced pain worsening on eye movements two weeks after receiving the Pfizer vaccine against COVID-19. The daughter recognised this as the initial symptom of post-vaccination optic neuritis from her mother's history and immediately presented to the hospital where a swollen optic disc was seen and high dose treatment with corticosteroids was initiated instantaneously. Within hours from starting the cortic [file 417_2025_6805_Fig9_ESM.png]

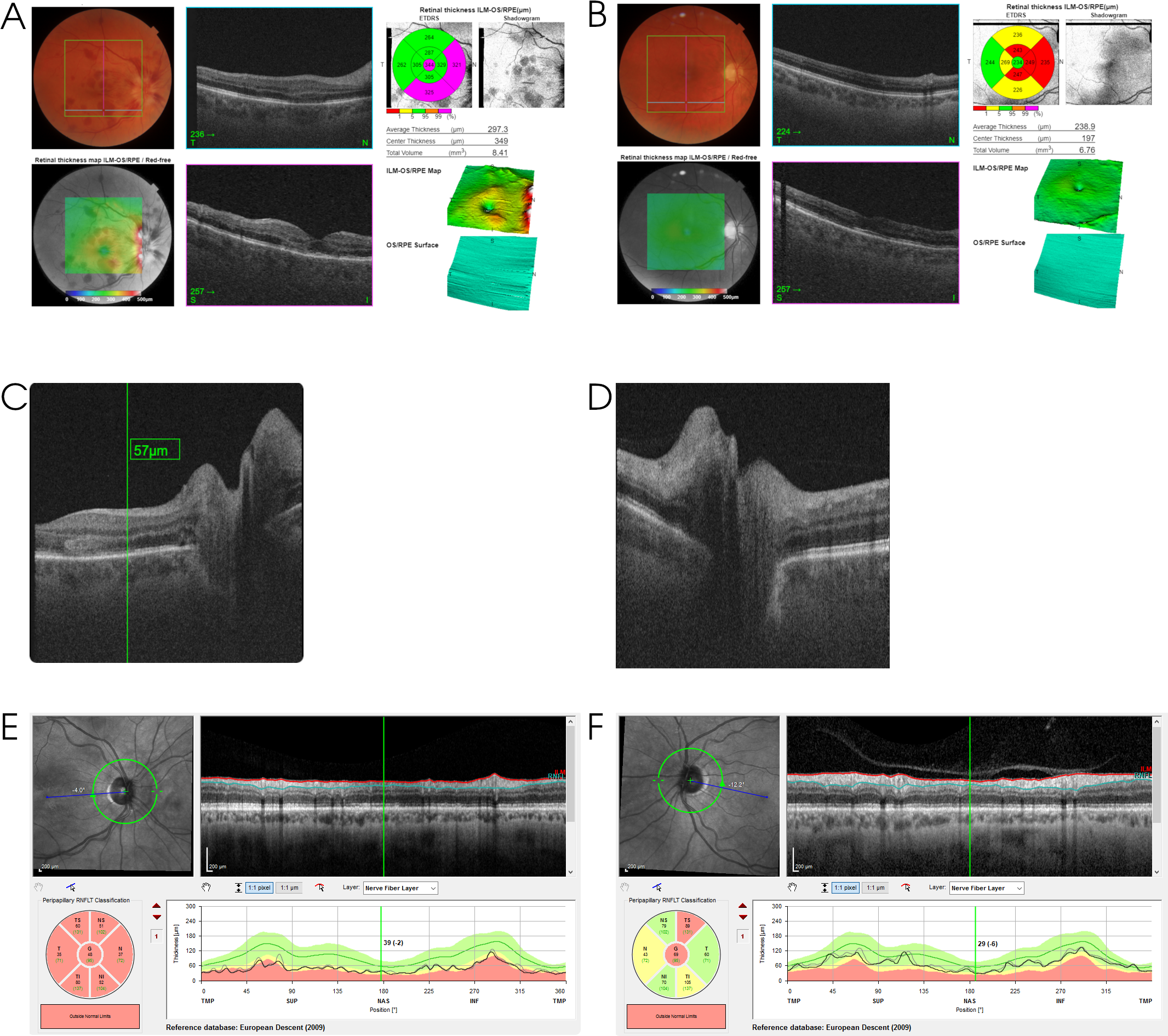

Supplement: Supplementary file 10 — Supplementary file5 (TIFF 11812 KB) [file 417_2025_6805_MOESM5_ESM.tiff]
