# Supplementary material for: Treatment of post-vaccination optic neuritis: implications from the global SARS-CoV-2 vaccination effort
Source: Graefes Arch Clin Exp Ophthalmol. 2025 Nov 29;264(3):851–62. doi: 10.1007/s00417-025-06805-w (PMC12966182; doi:10.1007/s00417-025-06805-w)
Supplement: Supplementary file 11 — Supplementary file6 (DOCX 60 KB) [file 417_2025_6805_MOESM6_ESM.docx]

Supplementary table 1: Summary of the 9 APOSTEL 2.0 items.

| Item | Description |
| --- | --- |
| 1 | 60 OCT devices from 59 sites in 55 countries. Baseline OCT at same time as clinical assessment and within one months of MRI. Follow-up OCT within one year. Exclusion criteria for OCT based on OSCAR-IB. |
| 2 | Three manufactures: Heidelberg Spectralis, Zeiss Cirrus, Topcon 1000/Triton. The device type is Spectral domain for Heidelberg and Zeiss. The device type is Swept-source for Topcon. |
| 3 | Pupils were not dilated and there was one operator per side. |
| 4 | The scanning protocol included a circular scan around the optic disc (Heidelberg), or extraction of such a circular scan from a volume scan of the optic disc (Zeiss, Topcon). A volume scan from the macula with the eye tracking function enabled for Heidelberg. |
| 5 | Fundus photography Topcon TRC-50DX. |
| 6 | OSCAR-IB. There was no eye selection strategy, we included both eyes because we also calculated the inter-eye difference. |
| 7 | Device proprietary segmentation and extraction of retinal layer thickness for the peripapillary RNFL, macular GCL, macular IPL. Calculation of the combined ganglion and inner plexiform layers (mGCIPL). The EDTRS 1,3,5 mm grid was used and the central area excluded from analyis. |
| 8 | Nomenclature compatible with Figure 1 in APOSTEL 2.0 guidelines. |
| 9 | Non-parametric regression (Spearman) and general linear models (GLM). Data were analysed per eye and per patient. |

**Supplementary table 2**: Overall MRI analyses for UK patients.

Twenty-four UK patients’ MRI scans analysed. MRI Brain and orbits were performed for all patients. a) Field strength in Tesla. b) Contrast given - Was iv gadolinium administered for the MRI scan. c) MRI affected side - radiological diagnosis for the affected optic nerve(s). d) Clinical side - clinical diagnosis for affected optic nerve(s). e) Optic nerve contrast enhancement - was this seen on either side. f) Optic nerve swelling - was this seen on either side. g) Brain - findings for pathological meningeal enhancement, presence of inflammatory/demyelinating lesion. BL=bilateral, PV = periventricular, JC = juxtacortical, IT = Infratentorial, L=left, N=no, NA=not available, R=right, Y=yes. h) Spine - presence of spinal lesions. NA = not applicable because not performed. Note that the first column refers to the study number of each case and is not sequential in this supplementary table.

| **UK**  **Case** | **Field Strength [T]** | **Contrast given?** | **MRI**  **affected side** | **Clinical side** | **Optic nerve contrast enhancement?** | **Optic nerve swelling**  **?** | **Brain** | | |
| --- | --- | --- | --- | --- | --- | --- | --- | --- | --- |
|  |  |  |  |  |  |  | Meningeal enhancement | Inflammatory lesions? | Comments |
| 1 | 1.5 | Y | BL | BL | N | Y | N | 1 | Enhancing midbrain lesion |
| 2 | 1.5 | Y | BL | BL | N | N | N | N |  |
| 3 | 1.5 | Y | BL | BL | Y | Y | N | N |  |
| 10 | 1.5 | Y | R | R | N | Y | N | >10 | PV, JC, IT |
| 11 | 1.5 | N | R | R | Not given | Y | NA | N |  |
| 12 | 1.5 | Y | L | L | Y | Y | N | N |  |
| 13 | 1.5 | N | L | L | Not given | Y | NA | >10 | PV, JC, IT |
| 14 | 1.5 | Y | L | L | N | N | N | >10 | PV, JC |
| 15 | 3 | Y | L | L | Y | Y | N | >10 | PV, JC |
| 16 | 1.5 | Y | R | R | Y | Y | N | 6 | PV, JC, |

| 17 | 3 | Y | L | L | Y | N | N | N |  |
| --- | --- | --- | --- | --- | --- | --- | --- | --- | --- |
| 19 | 3 | N | BL | R | Not given | Y | NA | N |  |
| 32 | 1.5 | Y | L | L | Y | Y | N | N |  |
| 33 | 1.5 | Y | L | L | Y | Y | N | N |  |
| 34 | 1.5 | Y | L | L | Y | Y | N | N |  |
| 35 | 1.5 | Y | BL | BL | Y | Y | N | N |  |
| 36 | 1.5 | Y | BL | BL | Y | Y | N | N |  |
| 37 | 1.5 | Y | R | R | Y | N | N | N |  |
| 38 | 1.5 | Y | L | L | Y | Y | N | N |  |
| 39 | 1.5 | Y | L | L | Y | Y | N | N |  |
| 40 | 3 | Y | BL | BL | Y | Y | N | 1 | Large left temporal lesion with oedema - possible rim enhancement |
| 41 | 1.5 | Y | BL | BL | Y | Y | N | N |  |
| 47 | 1.5 | Y | R | R | Y | Y | N | N |  |
| 48 | 1.5 | Y | negative | R | N | N | N | N |  |

**Supplementary Table 3.** MRI UK analysis for right orbit MRI

Right orbital findings for twenty four UK cases with MRI scans. a) STIR lesion length in mm. If blank then no lesion observed. b) GAD lesion length in mm. If blank then no lesion observed. c) Swelling - Was there segmental swelling along the hyperintense nerve d) Compartments involved for optic nerve lesion - 1=orbit, 2=canal,3=cranial, 4=chiasm. d) Sheath enhancement - Present or absent. e) EOM - Enlarged or enhancing extraocular muscles seen. f) Orbital fat - Involvement of orbital fat inflammation/stranding. g) SOF - Soft tissue involvement of superior orbital fissure. h) Lacrimal gland - enlarged/abnormally enhancing lacrimal gland, GAD=gadolinium.

| **UK Case** | **Right Optic Nerve** | | | | **Right Orbital Adnexa** | | | |
| --- | --- | --- | --- | --- | --- | --- | --- | --- |
|  | STIR lesion length | GAD lesion length | Swelling? | Compartments involved | Sheath enhancement? | EOM? | Orbital fat? | SOF? |
| 1 | 9.9 |  | Y | 1 | N | N | Y | Y |
| 2 | 12 |  | N | 1,2 | N | N | N | N |
| 3 | 42.9 | 36.6 | Y | 1,2,3,4 | Y | Y | Y | N |
| 10 | 16.5 |  | Y | 1 | N | N | Y | N |
| 11 | 12 |  | Y | 1 | NA | N | N | N |
| 12 |  |  | N |  | N | N | N | N |
| 13 |  |  | N |  | NA | N | N | N |
| 14 |  |  | N |  | N | N | N | N |
| 15 |  |  | N |  | N | N | N | N |
| 16 | 18.9 | 18.9 | Y | 1 | Y | N | N | N |

| 17 |  |  | N |  | N | N | N | N |
| --- | --- | --- | --- | --- | --- | --- | --- | --- |
| 19 | 9.3 |  | Y | 1 | NA | N | N | N |
| 32 |  |  | N |  | N | N | N | N |
| 33 |  |  | N |  | N | N | N | N |
| 34 |  |  | N |  | N | N | N | N |
| 35 | 25.5 | 26.4 | Y | 1,2 | N | N | N | N |
| 36 | 6.6 | 6 | Y | 1 | N | N | N | N |
| 37 | 10.8 | 8.8 | N | 1,2 | N | N | Y | Y |
| 38 |  |  | N |  | N | N | N | N |
| 39 |  |  | N |  | N | N | N | N |
| 40 | 10.8 |  | Y | 1 | N | N | N | N |
| 41 | 21 | 21 | N | 1 | Y | N | Y | N |
| 47 | 40 | 32 | Y | 1,2,3,4 | Y | N | Y | N |
| 48 |  |  | N |  | N | N | N | N |

**Supplementary Table 4.** MRI UK analysis for left orbit MRI

Left orbital findings for twenty-four UK cases. a) STIR lesion length in mm. If blank then no lesion observed. b) GAD lesion length in mm. If blank then no lesion observed. c) Swelling - Was there segmental swelling along the hyperintense nerve d) Compartments involved for optic nerve lesion - 1=orbit, 2=canal,3=cranial, 4=chiasm. d) Sheath enhancement - Present or absent. e) EOM - Enlarged or enhancing extraocular muscles seen. f) Orbital fat - Involvement of orbital fat inflammation/stranding. g) SOF - Soft tissue involvement of superior orbital fissure?. h) Lacrimal gland - - enlarged/abnormally enhancing lacrimal gland.

| **UK Case** | **Left Optic Nerve** | | | | **Left Orbital Adnexa** | | | | |
| --- | --- | --- | --- | --- | --- | --- | --- | --- | --- |
|  | STIR lesion length | GAD lesion length | Swelling? | Compartments involved | Sheath enhancement? | EOM? | Orbital fat? | SOF? | Lacrimal gland? |
| 1 | 13.2 |  | Y | 1 | Y | N | Y | Y | Y |
| 2 | 4 |  | N | 2 | N | N | N | N | N |
| 3 | 19.8 | 16.5 | Y | 1 | Y | N | Y | N | N |
| 10 |  |  | N |  | N | N | N | N | N |
| 11 |  |  | N |  | NA | N | N | N | N |
| 12 | 40 | 40 | Y | 1,2,3 | Y | N | Y | Y | N |
| 13 | 20 |  | Y | 1 | NA | N | Y | Y | N |
| 14 | 4 |  | N | 2 | N | N | N | N | N |
| 15 | 13.8 | 13.8 | Y | 2,3 | N | N | Y | N | N |

| 16 |  |  | N |  | N | N | N | N | N |
| --- | --- | --- | --- | --- | --- | --- | --- | --- | --- |
| 17 | 8 | 4 | N | 2 | N | N | N | N | N |
| 19 | 9.3 |  | Y | 1 | NA | N | N | N | N |
| 32 | 21.6 | 30.8 | Y | 1,2 | N | N | N | N | N |
| 33 | 30.8 | 17.6 | Y | 1 | Y | N | Y | Y | N |
| 34 | 21.6 | 22 | Y | 1 | Y | N | N | N | N |
| 35 | 25.2 | 26.4 | Y | 1,2 | N | N | N | N | N |
| 36 | 6.6 | 6 | Y | 1 | N | N | N | N | N |
| 37 |  |  | N |  | N | N | N | N | N |
| 38 | 28.8 | 17.6 | Y | 1,2 | Y | N | N | N | Y |
| 39 | 26.4 | 26.4 | Y | 1,2,3,4 | N | N | N | N | N |
| 40 | 25.2 |  | N | 1,2,3 | N | N | N | N | N |
| 41 | 24.78 | 24.78 | Y | 1 | Y | N | Y | N | N |
| 47 |  |  | N |  | N | N | N | N | N |
| 48 |  |  | N |  | N | N | N | N | N |

Supplementary Table 4: Corticosteroid Treamtment detailing the route of application, dosage and duration of treatment. The breakdown for cases with MOG is also provided because in MOG-ON this has prognostic implications ^30^. All patients with MOG-ON received high dose corticosteroids. Intravenous = IV, per oral = PO.

| **Corticosteroids** | **Entire cohort** | **MOG-ON** |
| --- | --- | --- |
| ‍Dosage (IV/PO as total number) |  | 0/0 |
| ‍ 0.4 g | 0/1 | 0/0 |
| ‍ 0.5 g | 2/2 | 0/0 |
| ‍ 0.75 g | 0/1 | 0/0 |
| ‍ 1.0 g | 44/3 | 11/2 |
| ‍ 1.25 g | 0/7 | 0/2 |
| ‍Duration (days) |  |  |
| ‍ 3 | 23 | 7 |
| ‍ 4 | 2 | 1 |
| ‍ 5 | 27 | 7 |
| ‍ 7 | 5 | 0 |
| ‍ 8 | 1 | 0 |
| ‍ 10 | 1 | 0 |
| ‍ 35 | 1 | 0 |
